# Ga-Based Liquid Metal Catalyst for Mechano-Assisted Carbon–Carbon Coupling Reaction

**DOI:** 10.34133/research.1099

**Published:** 2026-01-27

**Authors:** Pengkun Yang, Zijuan Hu, Qingyu Wang, Binqian Liu, Mengyang Cao, Lu Huang, Peng Liang, Yingpeng Wu

**Affiliations:** ^1^ State Key Laboratory of Chemo and Biosensing, College of Chemistry and Chemical Engineering, Advanced Catalytic Engineering Research Center of the Ministry of Education, Hunan University, Changsha, 410082, P. R. China.; ^2^ Department of Chemistry, The University of Hong Kong, Hong Kong 999077, China.

## Abstract

Traditional solid metal catalysts in organic reaction have been hindered by drawbacks, including limited surface catalytic region, restricted reactant diffusion, catalyst deactivation, low atom utilization efficiency, and harsh reaction condition. To overcome these drawbacks, we introduce liquid metal into organic reaction. An efficient mechano-assisted reaction system was proposed integrating dynamic metal catalyst and liquid metal reaction medium. With a low-energy ball-milling process (at a speed of 270 rad·min^−1^), the dynamic metal atoms in liquid state could achieve maximum atomic utilization and fast reaction kinetics compared to solid metal. Furthermore, liquid metal also act as reaction media. It not only offers a homogeneous electron-rich environment for reactants but also continuously disperses active metals to construct a multimetal catalytic system in one step. These would revolutionize the understanding of metal catalysts and reaction media, providing a novel platform for exploration of high-throughput catalysis and discovery of novel chemical reaction in future.

## Introduction

Metal catalysts, typically in solid state, play a central role in the crossing-coupling reactions for the production of pharmaceuticals, energy storage materials, and other advanced functional materials [1–5]. Limited by the solid metal particle agglomeration and inefficient mass transfer at interfaces, low atomic utilization and catalyst poisoning remain formidable challenges for solid metal catalysts. In recent years, the use of ball-milling technique has reignited marked interest in metal catalysts, such as lithium [6], calcium [7], magnesium [8], manganese [9], palladium [10], etc. Compared with the traditional metal-catalyzed reaction, the ball-milling technique provides a simple, efficient, and eco-friendly alternative [11,12]. Without additional steps and harmful solvents, the entire reaction process can be achieved solely through mechanical energy, including the removal of the oxide layer to activate the metal, the pulverization of metals to expose additional catalytic sites, and the promotion of the mass transfer between catalysts and reactants. However, these processes typically rely on a high-energy ball milling (mostly ≥1,200 rad·min^−1^), resulting in high costs for equipment procurement and maintenance that hinder its further application.

As an emerging material, gallium-based room-temperature liquid metals (Ga-LMs) with combined characteristics of metal and fluid have been brought out breakthroughs in the fields of electronic engineering [13], additive manufacturing [14,15], biomedicine [16–18], intelligent robot [19,20], and synthesis for functional materials [21–24]. Recently, some scientific researchers have also discovered that the Ga-LMs with dynamic fluidity could markedly enhance the reaction kinetics, eliminate the drawbacks of coking or coarsening [25–27], and exhibit superior catalytic activity and stability over conventional solid metal catalysts [28–31]. In addition, different from traditional ionic or molecular environment, the Ga-LMs can offer a unique liquid reaction environment with electron-rich and dynamic catalytic sites, which is very attractive for discovery of novel chemical reactions and exploration of highly efficient catalyst systems. Regrettably, research on liquid metal catalysts in organic reactions is still rarely reported, primarily impeded by 3 key factors: (a) the low wettability of liquid metal with organic reactants results in the catalytic region being restricted to surface interactions [32], losing the benefits of liquid metal fluids. (b) The limited liquid metal catalyst varieties constrain their widespread application in organic reactions [33]. (c) Although liquid gallium could dissolve or disperse metals to form composite catalytic systems [34], the trace active metals dispersed in gallium are not sufficient for sustainable reaction. Given the promising attributes and advantages of liquid metal catalysts, exploring their application in organic synthesis holds significant potential and challenges.

Building on these considerations, we propose a strategy of mechano-assisted liquid metal catalytic system and experimentally demonstrate its exceptional efficiency in mediating carbon–carbon bond formation. Driven by lower mechanical energy (revolution speed at 270 rad·min^−1^), the organic reactants could overcome the surface tension of liquid metal catalyst, being homogeneously dispersed in the liquid metal phase. In parallel, such catalysts can expose and renew dynamic catalytic active sites, achieving maximum utilization of metal atom and higher product yield. Not only that, the active metal created by mechanical force could be continuously dissolved and dispersed in liquid gallium. On the basis of this catalytic system, we explore liquid metal catalysts in Ullmann-type reductive coupling reaction and [2 + 2 + 2 + 2] cycloaddition of alkynes, providing a detailed explanation of their advantages over solid metals in terms of energy dissipation, reaction mass transfer, and electronic structure. In addition, the obtained conjugated porous organic polymer (CPOP) from small-molecule coupling exhibits immense potential for the cathode of Na/Cl_2_ battery. On the whole, this strategy not only has the advantages of operationally simple, efficiency, low mechanical energy consumption, and universality but also presents a brand new concept of electron-rich liquid metal catalytic systems for organic chemistry.

## Results and Discussion

### Characteristics of mechano-assisted liquid metal catalytic system

**Fig. 1.**
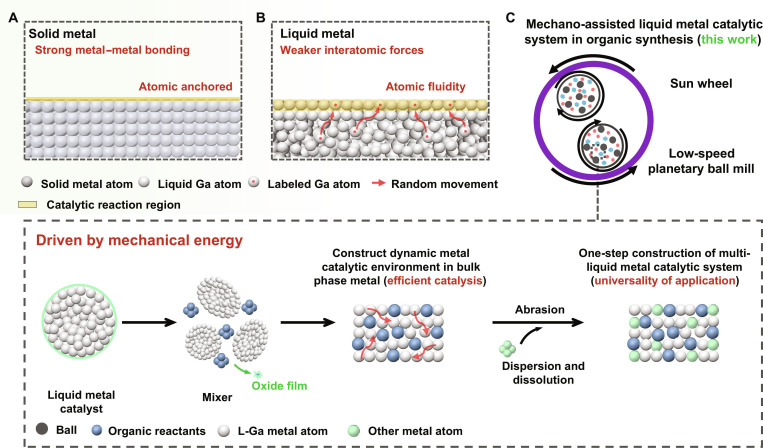
Characteristics of the solid–liquid metal reaction interface and mechanically assisted liquid metal catalytic systems. (A and B) Schematic diagram of atomic motion in solid and liquid metal catalytic processes. (C) Schematic diagram of mechanical ball-milling-assisted liquid metal catalysis system.

One goal in catalysis research is the design of highly efficient catalysts with a larger number of active sites. For solid metal catalysis, it is extremely challenging, as the solid catalyst only offers surface active sites, while the interior atoms contribute minimally. In addition, the active sites anchored on the catalyst surface are easily covered and deactivated by generated products (Fig. 1A). As a comparison, the liquid metal catalyst with the high mobility of the surface atoms could constantly offer more fresh reaction sites to markedly increase the reaction efficiency (Fig. 1B). On this basis, we built a class of sustainable dynamic metal catalytic systems by ball-milling technique (Fig. 1C and Fig. S2). In this system, the high surface tension (e.g. gallium ≈ 700 mN·m^−1^ at 30 °C) can be overcame, and the liquid metal is easy to break apart into small units and expose more fresh interfacial catalyst sites under mechanical energy. Meanwhile, the reactants could get inside liquid metal that is consisting of a “sea” of free electrons, and the dynamic metal atom and rich electrons would construct a catalytic environment integrating homogeneous metal-based catalysts and media synergistically for reactants. Besides, when other solid catalytically active metals are introduced into the reaction system, they can dissolve and disperse in liquid gallium under mechanical attrition, constructing a multiliquid metal catalytic system for diverse organic reactions.

### Liquid-gallium-metal-catalyzed C–C coupling of small molecules

**Fig. 2.**
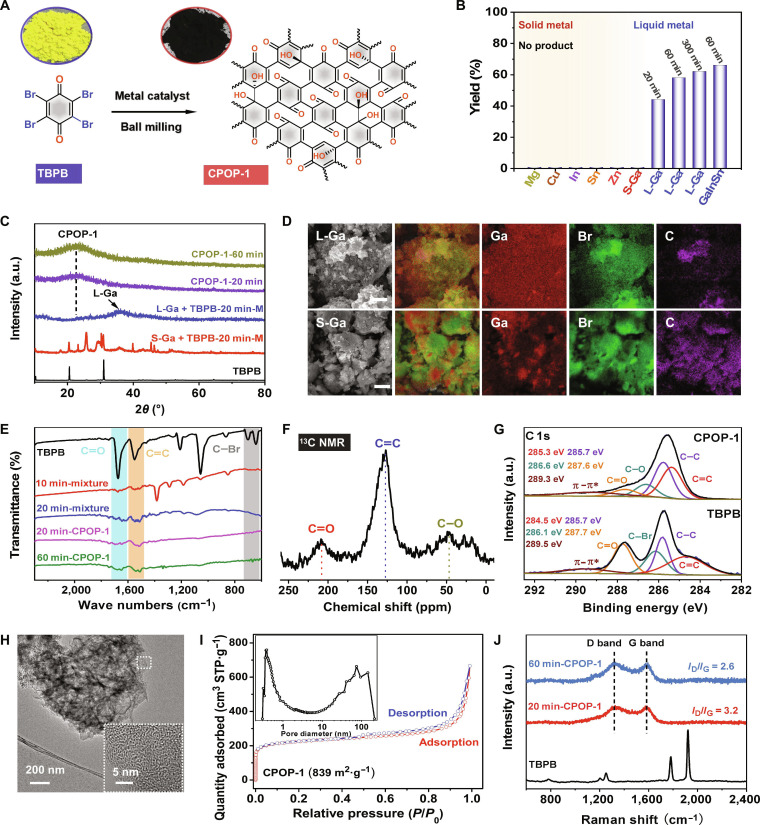
Characterization of liquid-gallium-catalyzed TBPB monomer coupling. (A) Reaction equation for the synthesis of CPOP-1 (black powder) from TBPB monomer (light yellow powder) via mechanochemistry. (B) The yield of CPOP-1 catalyzed by different metal catalysts (All metal catalysts were added 4 equiv; detail information in the Supplementary Materials). (C) XRD patterns of TBPB, mixtures catalyzed by liquid Ga and solid Ga (the “M” denotes a mixture in the figure), and CPOP-1 products with different reaction times. a.u., arbitrary units. (D) SEM and EDS images of mixtures catalyzed by liquid Ga and solid Ga. Scale bar, 5 μm. (E) IR absorption spectra of TBPB, mixtures catalyzed by liquid Ga with different reaction times, and CPOP-1 products with different reaction times. (F) Solid-state ^13^C NMR spectrum of CPOP-1 product obtained after ball milling for 1 h. (G) C 1s XPS of TBPB monomer and CPOP-1 obtained after ball milling for 1 h. (H) TEM images of CPOP-1 product obtained after ball milling for 1 h. (I) N_2_ isotherms and pore size distribution curves (inside) of CPOP-1 obtained after ball milling for 1 h, STP (standard temperature and pressure). (J) Raman spectrum of TBPB and CPOP-1 products at different ball-milling times.

To demonstrate the advantages of mechano-assisted liquid metal catalytic system, we specially chose solid 2,3,5,6-tetrabromo-1,4-benzoquinone (TBPB) as the template reaction for C–C coupling (Fig. 2A) and compared the catalytic effects across this system, mechano-assisted solid metal catalytic system, and traditional solution system. As shown in Fig. 2B and Table S1, under the same ball-milling conditions, no CPOP-1 product can be collected using solid Ga metal powders or other solid metal powders (Fig. S3), such as Mg, Zn, In, Sn, and Cu (entries 1, 10, 11, 12, 13, and 14 in Table S1). While the liquid Ga (melting point at 29.8 °C) could obtain 44% yield product in a very short time (20 min; entry 2 in Table S1), the yield of target product tended to be stable after 1 h (58%; entry 3 in Table S1). For the GaInSn alloy catalyst in the liquid state, it can also obtain 66% yield product after 1 h (entry 9 in Table S1). Besides, traditional organic solvent system cannot prepare the CPOP-1 product even after 600 min of reaction (entry 15 in Table S1). Those results reveal the superiority and uniqueness of the mechano-assisted liquid metal catalytic system.

Following these observations, we systematically investigated this catalytic system in monomer coupling procedure from physical morphological and chemical properties. Through the x-ray diffraction (XRD) analysis of the postreaction mixture and target product (Fig. 2C), a large amount of unreacted numerous monomer and solid gallium remain in the mixture catalyzed by solid Ga, while in the mixture catalyzed by liquid gallium, the peaks of TBPB monomer have entirely disappeared. This suggests that the liquid gallium is more advantageous for creating a homogeneous catalytic environment for facilitating coupling. Correspondingly, the scanning electron microscopy (SEM) images also illustrate this point from a microscopic perspective (Fig. 2D). Under mechanical force, the solid gallium metal powders and TBPB monomer aggregate tightly into larger particles and separate from each other, greatly reducing the catalytic contact area. In contrast, liquid gallium could act as liquid medium to coat or disperse within the reactants, maximizing catalytic contact sites.

In addition, with the aid of Fourier transform infrared (IR) spectrometer, x-ray photoelectron spectroscopy (XPS), and magic angle spinning nuclear magnetic resonance (MAS-NMR), we also investigated the cross-linking process of TBPB catalyzed by liquid gallium and proposed a reasonable reaction mechanism. As the ball-milling time increases, the C–Br (639 cm^−1^) bond of TBPB gradually disappears, and only C═C (1,545 cm^−1^) and C═O (1,675 cm^−1^) bonds remained (Fig. 2E). In parallel, the disappearance of C–Br (286.1 eV) bond and the appearance of Ga–Br (21.7 eV) bond can also be observed from the C 1s and Ga 3d spectra (Fig. 2G and Fig. S4F). Interestingly, the binding energy of Ga–O (533.4 and 19.4 eV) is observed in the O 1s and Ga 3d spectra when all operations were performed under anaerobic conditions (Fig. S4E and F). According to the above results and previous literature [35–37], we speculate that the synthesis of the CPOP-1 product may undergo a single-electron transfer pathway. The electron-rich gallium can activate C–Br and C═O bonds to generate carbon radicals and ketyl radical anions and the construction of a C–C bond via homocoupling of the 2 radicals, leading to the generation of the final product (Fig. S5). To prove this hypothesis, we performed a controlled experiment by adding a free-radical scavenger 2,2,6,6-tetramethyl-1-piperidinyloxy (TEMPO) into reaction system. In addition, no CPOP-1 product was obtained, while some kinds of product TBPB-TEMPO were detected by high-resolution mass spectroscopy (Fig. S6), aligning with the above proposed mechanism. Subsequently, the structure of CPOP-1 product was characterized. As shown in the MAS-NMR spectroscopy (Fig. 2F), 3 peaks located at 50, 129, and 209 parts per million (ppm) are assigned as C–OH groups, sp^2^-hybridized carbon and C═O groups, respectively. In addition, these functional groups were also confirmed in the C 1s and O 1s XPS spectra of CPOP-1 product, corresponding to the peaks at 285.3, 286.1, 287.7, 532.5, and 533.8 eV (Fig. 2G and Fig. S4B). Besides, the thermal weight loss of CPOP-1 can reach 47% at 1,000 °C (Fig. S7A). These results indicate that the chemical structure of CPOP-1 possesses a conjugated carbon–carbon structure with abundance of carbonyl and hydroxyl groups. Meanwhile, the cross-linking of 2 sites also leads to diversity of nanostructures. The SEM images of CPOP-1 product displayed a stack of coiled and stretched nanosheet structures (Fig. S7B). Transmission electron microscopy (TEM) images further confirmed the disordered structure of nanosheet and porous channel morphology (Fig. 2H). This is also consistent with the XRD patterns and N_2_ sorption isotherms. The CPOP-1 product has a broad diffraction peak at round 23°, corresponding to the (002) plane in amorphous carbon structure (Fig. 2C). In addition, it shows a high surface area (839 m^2^·g^−1^) and total pore volumes (1.27 cm^3^·g^−1^) with a broad pore size distribution, including micropores, mesopores, and macropores (Fig. 2I). Further quantitative measurement by Raman spectra showed that the defect degree of CPOP-1 decreases (the band area ratio *I*_D_/*I*_G_ from 3.2 to 2.6) with the extension of ball-milling time (Fig. 2J). That is, higher polymerization degree achieved by more cross-linking time led to more organized scaffolds with fewer defects. In general, electron-rich liquid gallium with dynamic catalytic sites exhibits superior catalytic performance to solid metals and offers an efficient alternative for solvent-free solid reaction systems.

### Expansion of multiliquid metal catalytic systems (L-GaMg and L-GaNi) and reactions

**Fig. 3.**
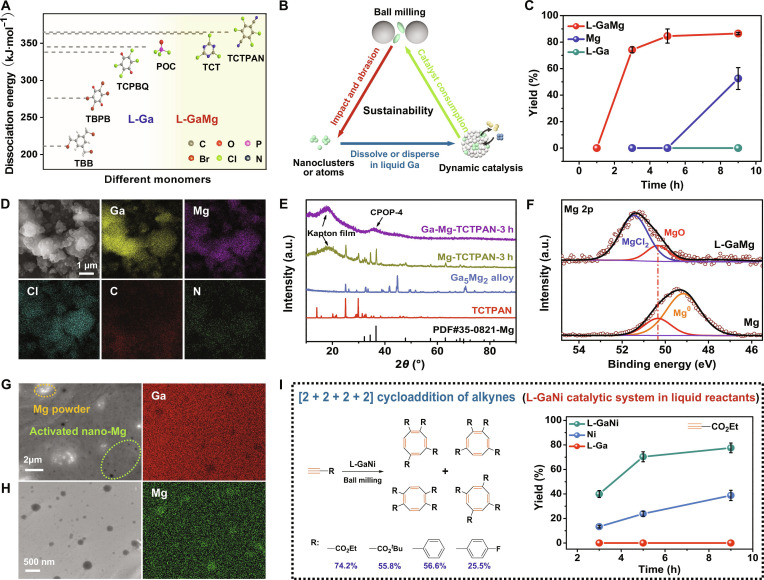
Characterization of liquid-GaMg- and liquid-GaNi-catalyzed molecular coupling. (A) The dissociation energy of different halogenated reactants. (B) Diagram of a sustainable dynamic metal catalytic reaction process. (C) TCTPAN monomer coupling reaction yields of catalyzed by Ga, Mg, and composite liquid GaMg catalytic system (error bars on yield represent the SD obtained from 3 measurements). (D) SEM and EDS images of mixtures catalyzed by composite liquid GaMg catalytic system. (E) XRD patterns of TCTPAN, Ga_5_Mg_2_ alloy obtained by ball-milling liquid gallium and Mg powder, and reaction mixture catalyzed by Mg powder and composite liquid GaMg catalytic system. (F) Mg 2p XPS spectra of the reaction mixture catalyzed by Mg powder and composite liquid GaMg catalytic system. (G and H) SEM and EDS images of composite liquid GaMg catalytic system. (I) [2 + 2 + 2 + 2] cycloaddition of alkynes catalyzed by gallium, nickel and composite liquid GaNi catalytic system (error bars on yield represent the SD obtained from 3 measurements).

The feasibility of our approach was studied using other monomers with different active halocarbon groups. As shown in Fig. 3A, 1,3,5-tris(bromomethyl)benzene (TBB) and the 2,3,5,6-tetrachloro-1,4-benzoquinone (TCPBQ) monomers can be catalyzed by liquid gallium to obtain the CPOP-2 and CPOP-3 product, correspondingly (Figs. S8 and S9). However, as the monomer’s C–X bond dissociation energy increasing (Table S2), the liquid gallium metal is not sufficient to activate the monomer for cross-linking reaction, such as phosphorus oxychloride (POC), tetrachloroterephthalonitrile (TCTPAN), and 2,4,6-trichloro-1,3,5-triazine (TCT). On the basis of the solubility characteristics of liquid gallium and single atom prepared by mechanical ball grinding, composite gallium-based liquid metal catalytic system with higher catalytic activity was achieved to address the aforementioned issues. As illustrated in Fig. 3B, under repeated mechanical collisions and friction, the solid metal undergoes deformation and fragmentation, as well as surface abrasion, leading to the generation of atomic-level particulates [38]. Then, these atomized metals will dissolve and disperse into the liquid gallium to a dynamic and liquid catalytic environment. Notably, these dispersed active metals can continue to regenerate incessantly under mechanical energy while consuming by reactants, thus ensuring the sustainable progression of reaction. This sustainable catalytic process cannot be achievable with currently reported composite liquid metal catalysts. Taking the coupling of TCTPAN monomer as an example, we investigated the catalytic properties of the L-Ga, Mg powder, and composite liquid GaMg catalytic system (Table S3). Compared with the pure L-Ga and solid Mg powder, the composite liquid GaMg catalytic system could obtain a higher yield in a shorter time (Fig. 3C), which is only 1/3 time of solid Mg metal. In the composite liquid GaMg catalytic system, liquid gallium could act as a “solvent” to disperse solid Mg metal and reactants (Fig. 3D), establishing a homogeneous reaction environment that address the issue of aggregation and sintering in solid Mg metal (Fig. S10). After 3 h of ball-milling reaction, no characteristic XRD peaks of Mg metal, Ga_5_Mg_2_ alloy, and TCTPAN monomer were observed in mixtures catalyzed by liquid GaMg (Fig. 3E), and the Mg 2p XPS spectrum shows that the Mg^0^ is completely converted to MgCl_2_ (Fig. 3F). In contrast, there is still a amount of raw materials and Mg^0^ metal in the solid Mg reaction system. These results indicate that the Mg metals in the liquid GaMg system exhibit superior reactivity compared to solid magnesium.

To gain further insight, we discovered active nano-Mg in ball-milled Mg metal with liquid gallium, which is consistent with our conjecture in Fig. 3B. As shown in the Fig. 3G and H, it is observable that many new Mg nanoparticles with 10 to 200 nm in diameters (dark regions) are floating and dispersing in a sea of liquid Ga (Fig. S11). In addition, the absence of characteristic peaks for Mg and Ga_5_Mg_2_ alloy in the XRD spectra indicates a stable and homogeneous dispersion of nano-Mg particles within the liquid gallium (Fig. S12). In addition, the higher metal activity of the liquid GaMg catalytic system can be proved by the more violently reaction with water than solid magnesium (Movie S1). As far as we know, this nanoscale active Mg with dynamic and high metal activity is a novel state and previously unidentified and unreported. In light of this, polymer CPOP-4 and CPOP-5 were also successfully synthesized by the self-coupling of TCTPAN and TCT monomers (Figs. S13 and S14) and even achieved dual-molecular coupling of TBPB and POC monomer to obtain the phosphorus-doped functional carbon polymer CPOP-6 (Fig. S15). Notably, the approach can also be extended to construct other composite liquid metal catalytic systems and remains effective for reaction systems with liquid reactants. As shown in Fig. 3I, we evaluated the catalytic performance of liquid GaNi catalytic system in [2 + 2 + 2 + 2] cycloaddition of alkynes (Table S4). Similarly, the liquid GaNi composite catalytic system with highly dispersed nickel catalytic sites (Figs. S16 and S17) has consistently exhibited higher catalytic efficiency (3 times) than solid Ni powders (Fig. 3I and Fig. S18). Even with other alkyne substrates, this catalytic system maintains exceptional selectivity and efficiency (Figs. S19 to S21). These findings demonstrate the efficiency and broad application potential of mechano-assisted liquid metal catalytic system in organic synthesis.

### Analysis of the advantages of liquid metal catalytic systems

**Fig. 4.**
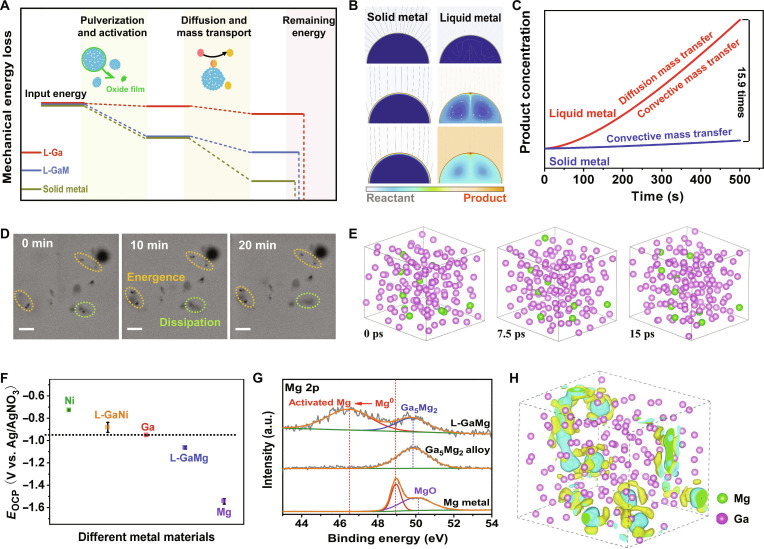
Analysis of high-efficiency catalysis in liquid metal catalytic systems. (A) The mechanical energy loss of different metal catalysts during ball-milling process. (B and C) Finite element simulation of mass transfer in solid and liquid metal catalytic process. (D) SEM images of dynamic movement of Mg dispersed in liquid gallium. Scale bars, 2.5 μm. (E) AIMD simulation of Mg atom motion in liquid gallium. (F) The OCP curves of different metals (error bars represent the SD obtained from 3 measurements). (G) Mg 2p XPS spectra of Mg dispersed in liquid gallium. (H) The 3-dimensional charge density for liquid GaMg. The gold color denotes charge accumulation, whereas the cyan color represents the charge depletion zone. The isosurface set a uniform value of 0.003 electrons·Å^−3^.

For the efficient catalytic mechanism of liquid metal catalytic system, we carried out an in-depth investigation from energy dissipation, reaction mass transfer, and electronic structure. According to the previous studies on mechano-assisted metal catalyst, the catalytic process of metal catalysts usually undergoes 4 processes: metal activation, reactant adsorption, intermediate conversion, and product desorption [7,39–41]. In addition, the mechanical energy serves as the prime driver in overcoming the energy barrier of a reaction and facilitating its progression. Figure 4A illustrates a schematic diagram of the catalytic process of solid–liquid metal catalyst and the allocation of mechanical energy consumption. Compared to the high-energy consumption during the activation of solid metals, liquid metal catalyst with low interatomic forces is easier to break apart into small units and expose more catalyst site at less mechanical energy (Fig. S22). Besides, the fluid nature of liquid metal catalyst not only ensures uniform distribution and consistent catalytic activity but also facilitates the rapid diffusion of reactants and products during ball milling, thereby overcoming the low diffusion rate of solid metal. These indicate that in the liquid metal system, the process of converting mechanical energy into chemical energy is more efficient.

Subsequently, the mass transfer process of solid and liquid metal catalysts through finite element simulations was also explored (Fig. 4B and Movie S2). Under the optimal conditions, the mass transfer of solid metals is limited in terms of surface diffusion and desorption, whereas in liquid metals, it involves surface diffusion, desorption, and internal convective flow processes. Under equal conditions, the mass transfer of liquid metals with mobilized atoms is approximately 16 times faster than solid metals with the relatively stationary atoms (Fig. 4C). Furthermore, the fluidic nature of liquid gallium also imparts dynamic catalytic properties to composite liquid metal catalytic system, effectively exposing numerous catalytic sites and preventing catalyst poisoning. As illustrated in Fig. 4D, the in situ SEM captures that the dynamic movement of nanosized metal particles can be observed in liquid gallium, which migrates between the bulk phase and interfaces (Movie S3). In addition, this observation is consistent with the dynamic atomic motion simulation in liquid metals (Fig. 4E and Movie S4). During ball-milling process, the catalytic sites would exhibit more intense movement driven by mechanical force in contrast to its static state, favoring the overall kinetics of a reaction.

During a systematic study, we found that the metals dispersed in liquid Ga could be activated by the surrounding Ga atoms, leading to a favorable electronic structure for reactions. The open-circuit potential (OCP) analysis of various metallic systems indicated a notable activity difference between the composite liquid metal and pure metals (Fig. 4F and Fig. S23), which was proven by XPS and simulations to result from electron transfer. As shown in Fig. 4G and Fig. S24, the electrons of Mg nanoparticles dispersed in liquid gallium will transfer to the gallium system and increase the electron density of the composite liquid GaMg system, which could correlate with the negative shift of Mg 2p to a lower binding energy (shifted by 2.5 eV), as observed by XPS spectrum. To provide a clearer illustration of this viewpoint, we studied the electron density distribution in a composite liquid metal system by density functional theory (DFT) and found that the presence of magnesium metal increased the electron cloud in the surrounding area (Fig. 4H). The electron-rich environment would provide an abundance of electrons for the electrophilic reactants dispersed in liquid metal, potentially leading to higher reaction rates for single-electron transfer reaction. Likewise, we studied the electronic structure of liquid nickel composite systems. The nickel dissolved in liquid gallium could gain electrons from the surrounding gallium atom to increase its own electron density, which could correspond to the positive shift of Ni 2p to a higher binding energy (Fig. S25A). In addition, the electron-rich Ni active sites (Fig. S25B) can enhance the interaction with the alkyne, thereby improving the selectivity and yield of cyclization of alkynes. These advantages of liquid metal break through the constraints of traditional solid metal catalysts, offering a novel metal catalytic system option for organic synthesis.

### Performance of synthetic functional material in Na/Cl_2_ battery

**Fig. 5.**
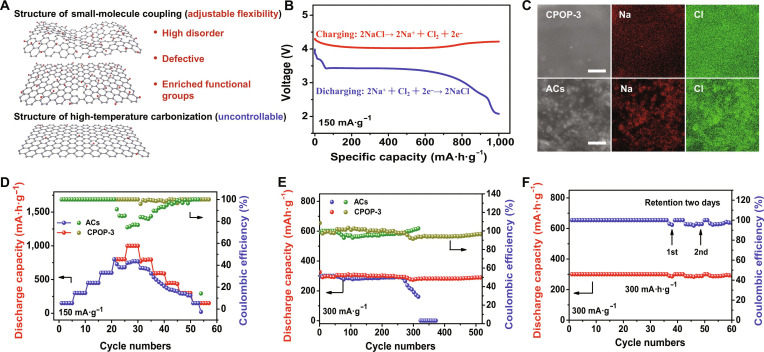
Performance of rechargeable Na/Cl_2_ battery featuring CPOP-3 and AC as the cathode. (A) Schematic diagram of the advantages of small-molecule coupling compared to high-temperature carbonization in the preparation of electrode materials. (B) Charge–discharge curve of the battery recorded at 1,000 mA·h·g^−1^ (150 mA·g^−1^). (C) SEM images of CPOP-3 and AC after the state of discharging from 800 mA·h·g^−1^. Scale bars, 5 μm. (D) Cycling performance of the Na/Cl_2_ battery at varying specific charging capacities from 150 to 1,000 mA·h·g^−1^ (150 mA·g^−1^). (E) Cycling performance of Na/Cl_2_ battery at a charging capacity of 300 mA·h·g^−1^ and a current density of 300 mA·g^−1^. (F) Cycling performance of the Na/Cl_2_ battery with different retention cycles at 300 mA·h·g^−1^ (300 mA·g^−1^).

Compared to materials produced by high-temperature carbonization, the small-molecule coupling method has more flexibility and advantages in constructing material structure (Fig. 5A). By selecting the appropriate molecules based on application requirement, it could impart many unique physicochemical properties to materials. As illustrated in Fig. S26, the XRD, IR, and electron paramagnetic resonance (EPR) analyses reveal that the CPOP-3 synthesized from TCPBQ molecules exhibits higher degrees of structural disorder, more defects, and a greater abundance of C═O and C═C bonds compared to active carbon (AC; specific surface area, 1,800 m^2^·g^−1^). These characteristics endow it as a promising candidate for the cathode material in rechargeable Na/Cl_2_ batteries. As shown in Fig. 5B, the Na/Cl_2_ battery redox process mainly entailed the transformation of NaCl and the capture of Cl_2_ during the process of charging and discharging [42–44]. In addition, the deposition state of NaCl crystals plays a critical role for the cycling durability of battery. Owing to the abundant functional groups, defects, and disordered porous structure in CPOP-3 material that facilitate the rapid uniform diffusion of Na^+^ [45–48], we found that the NaCl formed in CPOP-3 electrode is smaller and more uniformly distributed than AC electrode (Fig. 5C). This effectively inhibits the damage to electrode structure caused by the accumulation of NaCl crystals and enhances the conversion kinetics of 3-phase chlorine (NaCl/Na^+^ and Cl^−^/Cl_2_) [49]. On this basis, the CPOP-3 electrode exhibits excellent performance in both reversible capacity and cycle stability. For instance, the coulombic efficiency of CPOP-3 was maintained at ~100% when the capacities were increased from 150 to 1,000 mA·h·g^−1^, indicating the exceptional storage capacity of Cl_2_ gas and NaCl (Fig. 5D). In addition, it could maintain a long-term reversible capacity, reaching a capacity of 300 mA·h·g^−1^ at 300 mA·g^−1^ after 510 cycles, with the coulombic efficiency keeping around 97% (Fig. 5E). Even at a set specific capacity of 1,000 mA·h·g^−1^ at a current of 150 mA·g^−1^, it also can deliver 50 cycles (Fig. S28A). Notably, even after being shelved at open-circuit voltage for 2 days, the battery still maintains well-recharge ability, with a coulombic efficiency keeping around 97.5% (Fig. 5F and Fig. S28B). These results demonstrate the feasibility and potential of electrode material synthesized by small-molecule coupling in Na/Cl_2_ batteries.

## Conclusion

In this study, on the basis of the strategy of mechano-assisted liquid metal, we proposed a novel, solvent-free, and highly efficient dynamic metal catalytic system for C–C bond formation in small molecules. It overcomes key challenges including the low mass transfer efficiency and coking deactivation of solid metals, as well as the poor wettability of between liquid metal and organic reactants, enabling a catalytic liquid environment with self-renewing active sites and rich electrons. Thus, the coupling of TBPB molecules can be achieved by ball milling at 270 rad·min^−1^ for 20 min. Importantly, without additional processing, this strategy also can realize a one-step construction of diversified composite liquid metal catalytic system (L-GaMg and L-GaNi) at room temperature. The continuous generation of active metal in liquid gallium ensures the sustainability of reaction, while the diversity of composite liquid metal provides a basis for broadening their application to additional reactions. On the basis of these advantages, we synthesized a range of organic functional materials and investigated their performance in Na/Cl_2_ battery. The CPOP-3 material could maintain stable cycling for 510 cycles at capacity of 300 mA·h·g^−1^ at 300 mA·g^−1^, which is nearly double the cycle life of AC material. Overall, this work presents a new concept of dynamic metal catalysis with metal-based media, potentially setting off a new wave of research in the field of organic synthesis.

## Materials and Methods

### Materials

All the materials were obtained commercially, and no processing was done unless otherwise noted. Gallium metal (99.99%), indium particle (99%, 3 mm in size), tin powder (99%, 2 to 3 mm in size), magnesium powder (99%), copper powder (99%), zinc powder (98%), and nickel powder (99%) were purchased by Innochem Company. 2,3,5,6-tetrabromo-1,4-benzoquinone (98%), 1,3,5-tris(bromomethyl)benzene (98%), 2,3,5,6- tetrachloro-1,4-benzoquinone (98%), 2,4,6-trichloro-1,3,5-triazine (97%), tetrachloroterephthalonitrile (98%), phosphorus oxychloride (95%), aluminum chloride, ethyl propiolate (97%), *tert*-butyl propiolate (98%), phenylacetylene (97%), 4-fluorophenylacetylene (98%), ethyl alcohol, and ethyl acetate were obtained from Aladdin Company. Thionyl chloride (99%) was obtained from Macklin reagent. Ni foam (99.9%, 1 mm in thickness) was obtained from Taobao (power source battery sales department). Activated carbon-XFP01 was purchased from Nanjing XFNANO Materials Tech Co. Ltd. Sodium bis(fluorosulfonyl)imide (NaFSI; 99.9%) and sodium bis(trifluoromethylsulfonyl)imide (NaTFSI; 99.5%) were purchased from dodochem.com and were stored directly in the glove box.

### Material characterization

The planetary ball mill used in the experiment is Focucy FP-400, and milling jars with gas outlet/inlet valves were customized from Guangzhou Rurui Science & Technology Co. Ltd. The XRD data were recorded with a Rigaku Smartlab SE, and the diffraction patterns were recorded in the range of 10 to 90° at a scan speed of 5°·min^−1^. In addition, some air-sensitive samples are encapsulated by Kapton film in an inert glove box before testing. The near IR/IR absorbance curves were obtained using an IR Affinity-1 with a KBr in the range of 450 to 4,000 nm. Raman spectra were collected using a Witec Alpha300R with a 532-nm laser. The nitrogen adsorption isotherms were measured at 77 K by a BSD-660M A6B6M, the samples were outgassed at 150 °C for 12 h before the measurements, the surface areas were calculated from the adsorption data using Brunauer–Emmett–Teller methods, and the pore size distribution curves were obtained from the nonlocal DFT method. XPS analysis were carried on Thermo Fisher Scientific K-Alpha X (air atmosphere) and Thermo Fisher Scientific ESCALAB 250Xi (inert atmosphere). ^1^H NMR and ^13^C NMR spectra were carried out on a Bruker 400-MHz spectrometer with CDCl_3_ as the solvent. ^13^C direct polarization MAS (DPMAS) experiments were conducted using a Bruker Advance III 400WB spectrometer, equipped with a double resonance H/X CP-MAS 4-mm probe. The background signals originating from the probe’s static components were removed by the spin-echo sequence. The experimental conditions of ^13^C DPMAS were as follow: resonance frequency of 100.6 MHz, 90° pulse width of 2.5 μs, and delay time of 2s. Total acquisition time is 8 h. SEM and energy-dispersive spectrometer (EDS) were performed on MIRA3 TESCAN at 20 kV. TEM was conducted on an aberration-corrected FEI Titan G2 60-300. The defect information of materials has been confirmed by using EPR measurements, which have been obtained by Bruker EMXplus EPR spectrometer at room temperature. Galvanostatic discharge/charge measurements were performed on a NEWARE BTS-7.6.X battery program controlling system.

### General synthesis of CPOP products

Halogen elements were not detected in the characterized XPS spectrum of the CPOP series products. Therefore, yields of the CPOP products were calculated on the basis of the theoretical molecular weight after complete dehalogenation. The specific yield of CPOP products was calculated by the following equation and shown below.

$$\text{Yield of CPOP products}=\text{the mass of product}/n\left(M_{\text{monomer}}-{\mathrm{AM}}_{\text{halogen}}\right)$$ (1)

where *n* is the quantity of added material, $M_{\text{monomer}}$ is the molar mass of monomer, *A* is the number of halogens contained within monomer, and $M_{\text{halogen}}$ is the molar mass of halogen.

Halogen-containing molecule monomers, liquid gallium, and magnesium powder, 4 ZrO_2_ balls (Ø 10 mm), 14 ZrO_2_ balls (Ø 5 mm), and 14 ZrO_2_ balls (Ø 2 mm) were added to a 25-ml ZrO_2_ milling jar with gas outlet/inlet valves. After the jar was closed with purging inert gas, the reactor was ball milled in a planetary ball mill device at 270 rad·min^−1^ (revolution speed). Upon completion of the reaction, the mixture was thoroughly washed with 1 M HCl, deionized water, ethanol, and ethyl acetate for several times and dried at 60 °C under vacuum for 12 h. The reaction time and added catalyst vary with the halogen-containing molecule used, as detailed in the Supplementary Materials.

### General synthesis of [2 + 2 + 2 + 2] cycloaddition of alkynes

Nickel metal exhibits high selectivity in catalyzing the tetramerization of alkyne substrates and effectively suppressing the formation of trimeric by-products (Fig. S1). A total of 6 mmol of alkyne substrates, 7.2 mmol of Ni powder, and 5 mmol of liquid gallium, 4 ZrO_2_ balls (Ø 10 mm), 20 ZrO_2_ balls (Ø 5 mm), and 20 ZrO_2_ balls (Ø 2 mm) were added to a 25-ml ZrO_2_ milling jar with inert gas. After ball milling, the reaction mixture was extracted by addition of CH_2_Cl_2_, followed by centrifugal washing to collect the supernatant, purification via column chromatography. Further details are in the Supplementary Materials.

### OCP measurement

All electrochemical tests were conducted under ambient conditions, using a 3 electrode system on CHI 760e electrochemical workstation. A platinum wire was utilized as a counter electrode, a silver/silver nitrate reference (0.1 M AgNO_3_ in acetonitrile) served as a reference, and different metal samples were used as working electrode. The electrolyte was 0.1 M tetrabutylammonium hexafluorophosphate in *N*,*N*′-dimethylformamide solvent and was purged with nitrogen for 20 min before each measurement. In addition, the pure nickel and magnesium metal samples were ground with emery papers to remove the surface oxide film. The L-GaMg sample was obtained by ball mill for 0.5 h (1 g of gallium, six 3-mm-diameter Mg particles, and five 10-mm-diameter balls), while the L-GaNi sample was prepared by ball milling for 1 h (1 g of gallium, three 8-mm-diameter Ni beads, and five 10-mm-diameter balls). All samples should be transferred immediately to the electrochemical cell for testing after preparation. The data were measured 3 times to ensure the accuracy of results.

### DFT modeling and simulation of charger density distributions

All calculations were performed using the DFT, as implemented in the Vienna ab initio simulation package [50]. The projector augmented wave (PAW) method and Perdew–Burke–Ernzerhof generalized gradient approximation were used for the exchange correlation functionals . The Γ-point was used to sample the Brillouin zone of the supercell, and the time step was set to 2 fs. The canonical (NVT [number of particles, volume, and temperature]) ensemble with the Nose–Hoover thermostat was applied to control the temperature and the pressure in AIMD (Ab Initio Molecular Dynamics) simulations. The initial supercells of GaMg and GaNi contain 154 atoms each, with 10 for Mg or Ni, respectively, and 144 for Ga. The amorphous models were fabricated using a melt-quenching process. In this process, the primitive models were first subjected to complete melting at 2,000 K to erase the crystalline memory. Subsequently, the models were cooled down to 300 K at a rate of 33.3 K·ps^−1^ and then relaxed to attain a stable liquid state. Finally, the trajectories of atoms were collected, while the models were maintained at 300 K for 30 ps. For the charge density calculations, the energy cutoff of the PAW basis was set to 450 eV with a force convergence of 0.02 eV, and a 2 × 2 × 2 *k*-point grid was selected for the Brillouin zone sampling.

### Preparation and testing of Na/Cl_2_ battery

The circular Ni foam substrates with a diameter of 1.2 cm were sonicated in acetone and ethanol for 15 min, followed by drying in a 70 °C vacuum oven until the complete evaporated of all ethanol and measuring the weight of Ni foam substrates (1 mm in thickness). A total of 70% CPOP-3, 20% by weight of Super P, and 10% polytetrafluoroethylene (PTFE) (60% aqueous dispersion) were mixed in ethanol solvent. After ultrasonification for 2 h, the mixture was slowly brushed onto the Ni foam substrates. A loading range of 2 to 4 mg·cm^−2^ is desirable. The electrodes were dried overnight in a vacuum oven at 70 °C, and the weight of electrode was measured and calculated.

Prior to use, NaFSI and NaTFSI were dried overnight in a vacuum oven at 70 °C and then stored in an argon-atmosphere glove box. The appropriate amount of thionyl chloride was added into a bottle, and then a certain amount of anhydrous aluminum chloride (4 M) was weighed and fully dissolved in the thionyl chloride. Then, the appropriate amount of NaFSI and NaTFSI (2 wt % of the total weight of aluminum chloride and thionyl chloride) was added to the solution, followed by stirring until complete dissolution was achieved.

The assembly of all battery was conducted inside an argon-atmosphere glove box. Sodium metal block was removed the kerosene and oxide film on the surface, then cut to a suitable size, and pressed it on the 0.5-mm spacer by a tablet press, serving as the negative electrode. CPOP-3 loaded on Ni foam was used as the positive electrode. Two-layer quartz fiber filter membrane with a diameter of 18 mm was used as the battery separator and dried overnight in a vacuum oven at 70 °C before each use. When assembling the battery, the CPOP-3-positive electrode was placed in the center of the positive shell of the SS316 button battery, and then 2 layers of quartz diaphragm were placed above it. After adding the electrolyte (150 μl) to wet the separators, the sodium negative electrode on the spacer was placed on top, with the Na foil facing the CPOP-3-positive electrode. A spring was then positioned above the spacer, followed by a PTFE foil layer between the spring and the SS316-negative coin cell case to prevent electrolyte corrosion. Finally, the fully assembled coin cell was pressed at 50 MPa using a tablet press. The structural schematic diagram can be seen in Fig. S27. Then, the interstices along the edge of coin cell were encapsulated with a layer of EVA (ethylene-vinyl acetate copolymer) hot melt adhesive to prevent the ingress of water and air. After the EVA was cured, the battery was tested using a NEWARE BTS-7.6.X battery tester.

## Data Availability

All data needed to evaluate the conclusions in the paper are present in the paper and/or the Supplementary Materials.
