# Supplementary material for: Ga-Based Liquid Metal Catalyst for Mechano-Assisted Carbon–Carbon Coupling Reaction
Source: Research (Wash D C). 2026 Jan 27;9:1099. doi: 10.34133/research.1099 (PMC12935349; doi:10.34133/research.1099)
Supplement: Supplementary 1 — Supplementary Text Figs. S1 to S28 Tables S1 to S4 Movies S1 to S4 [file research.1099.f1.zip › Supplementary Materials.docx]

Supplementary Materials

**Ga-Based Liquid Metal Catalyst for Mechano-Assisted Carbon-Carbon Coupling Reaction**

Pengkun Yang^1^, Zijuan Hu^1^, Qingyu Wang^1^, Binqian Liu^1^, Mengyang Cao^1^, Lu Huang^1^, Peng Liang^2^* and Yingpeng Wu^1^*

^1^State Key Laboratory of Chemo and Biosensing, College of Chemistry and Chemical Engineering, Advanced Catalytic Engineering Research Center of the Ministry of Education, Hunan University, Changsha, 410082, P. R. China. ^2^ Department of Chemistry, the University of Hong Kong, Hong Kong, 999077, China.
* Address correspondence to: [wuyingpeng@hnu.edu.cn](mailto:wuyingpeng@hnu.edu.cn) (Y.W.); [liangp1@hku.hk](mailto:liangp1@hku.hk) (P.L.).

**This PDF file includes:**

Supplementary Text

Figs. S1 to S28

Tables S1 to S4

Movies S1 to S4

References

**Supplementary Text**

**Detailed synthesis procedures of CPOP products**

1 mmol TBPB monomer, 4 mmol liquid gallium, 4 ZrO_2_ balls (Ø 10 mm), 14 ZrO_2_ balls (Ø 5 mm) and 14 ZrO_2_ balls (Ø 2 mm) were added to a 25 ml ZrO_2_ milling jar with gas outlet/inlet valves. After the jar was closed with purging inert gas, the reactor was placed in a panetary ball mill device (Focucy FP-400, revolution speed at 270 rad/min). After grinding for 60 min, the reaction mixture was thoroughly washed with 1 M HCl, deionized water, ethanol and ethyl acetate for several times and dried at 60 °C under vacuum for 12 h, the black CPOP-1 product was obtained and calculated the yield (yield: 58%). CPOP-2 and CPOP-3 are synthesized in the same way as CPOP-1. The yellow CPOP-2 product was derived from 1 h ball milling of 1 mmol TBB monomer and 3 mmol liquid gallium (yellow powder, yield: 56.9 %), and the black CPOP-3 was prepared from 1h ball milling of 1mmol TCPBQ and 4 mmol liquid GaInSn alloy (black powder, yield: 52 %).

0.6 mmol TCTPAN monomer, 2.5 mmol magnesium powder, 5 mmol liquid gallium, 4 ZrO_2_ balls (Ø 10 mm), 14 ZrO_2_ balls (Ø 5 mm) and 14 ZrO_2_ balls (Ø 2 mm) were added to a 25 ml ZrO_2_ milling jar with inert gas. After ball milling for 5 h (270 rad/min), the reaction mixture was thoroughly washed with 1 M HCl, deionized water, ethanol and ethyl acetate for several times and dried at 60 °C under vacuum for 12 h, obtained black powder CPOP-4 and calculated the yield (black powder, yield: 84.6%). The procedure for CPOP-5 and CPOP-6 is the same as that for CPOP-4. 1 mmol TCT monomer, 3 mmol Mg powder and 5 mmol liquid gallium ball milling 9 h to obtain dark brown powder CPOP-5 (yield: 47.4%). And 0.6 mmol TBPB monomer, 0.4 mmol phosphorus oxychloride, 3.5 mmol Mg powder and 7 mmol liquid gallium were added to jar, we obtained black CPOP-6 powder after ball milling for 9 h (yield: 61.1%).

**Characterization for [2+2+2+2] cycloaddition of alkynes**

**
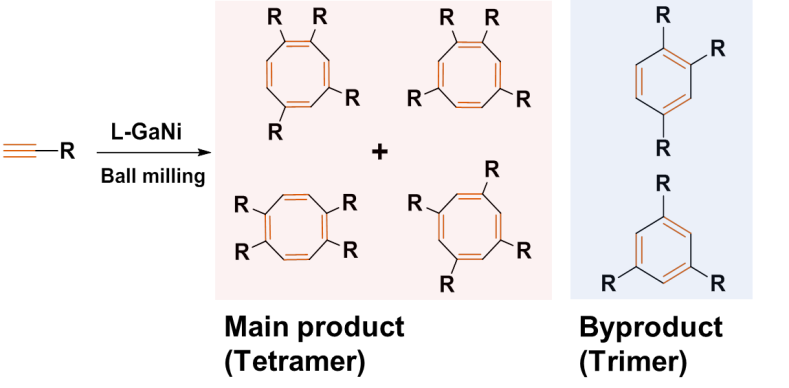
**

**Fig. S1.** [2+2+2+2] cycloaddition of alkynes.

The [2+2+2+2] cycloaddition of alkynes refers to a chemical reaction where four alkynes combine to form a cyclobutadiene derivative. It is characterized by the combination of multiple unsaturated molecules to form a cyclic structure. Usually, its products contain the main product of the tetramer and the byproduct of the trimer. Even with the selection of the appropriate metal catalyst, the product may still exhibit structural isomerism. Based on previous literature [[1](#_ENREF_1)], we have categorized these isomers as the primary products.


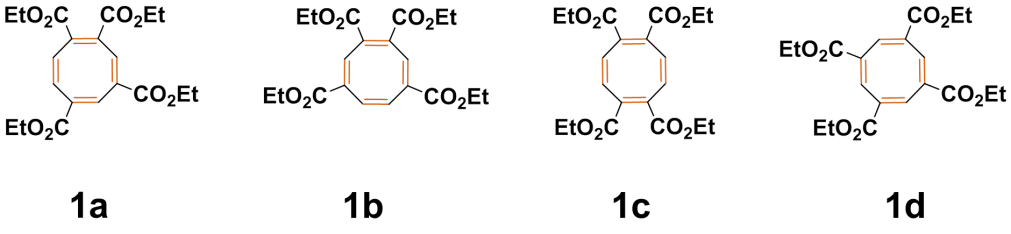


The title compound (**1a**-**1d**) was prepared by ethyl propiolate, and the yield of isomer mixture was 69.8% after ball milling for 5h. (yellow liquid product, 4:1 petroleum ether/ ethyl acetate). **^1^H NMR** (400 MHz, CDCl_3_, δ): 7.37, 7.35, 7.31, 7.29, 7.28, 7.20, 7.19, 7.15, 4.29, 4.28, 4.28, 4.27, 4.26, 4.26, 4.25, 4.24, 4.24, 4.23, 4.22, 4.22, 4.21, 4.20, 4.19, 4.17, 4.15, 4.14, 4.14, 4.13, 4.11, 1.32, 1.32, 1.31, 1.30, 1.30, 1.29, 1.28, 1.28, 1.27, 1.26, 1.25, 1.24, 1.23, 1.22, 1.21. **^13^C NMR** (101 MHz, CDCl_3_, δ): 164.71, 164.41, 164.35, 164.26, 141.04, 139.85, 139.78, 139.53, 139.42, 139.11, 134.68, 134.46, 133.71, 133.59, 133.29, 132.88, 61.82, 61.77, 61.63, 61.46, 61.39, 61.30, 14.26, 14.18. **MADLI-TOF-MS** (m/z) [M+Na]^+^ calcd for C_20_H_24_O_8_Na 415.1363, found 415.2820.


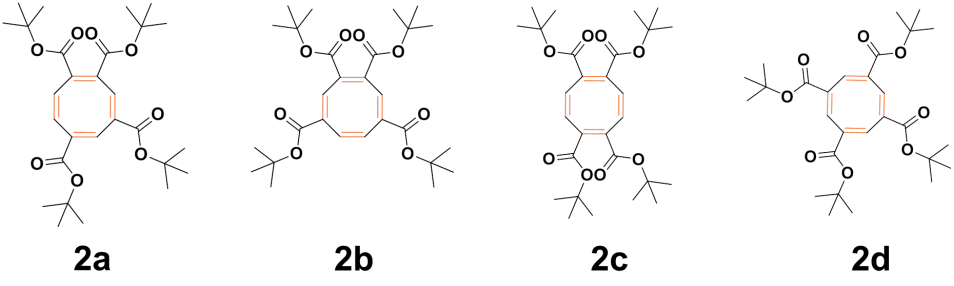


The title compound (**2a**-**2d**) was prepared by tert-butyl propiolate, and the yield of isomer mixture was 55.8% after ball milling for 5h. (light yellow powder, 4:1 petroleum ether/ ethyl acetate). **^1^H NMR** (400 MHz, CDCl_3_, δ): 7.17, 7.14, 7.11, 7.10, 6.99, 1.42, 1.38. **^13^C NMR** (101 MHz, CDCl_3_, δ) 164.12, 164.10, 164.06, 163.69, 163.65, 140.77, 140.44, 139.02, 138.99, 138.87, 138.61, 138.31, 137.97, 136.13, 135.96, 134.78, 134.66, 134.42, 134.03, 133.81, 82.30, 82.14, 82.08, 82.02, 81.77, 81.74, 81.40, 81.09, 28.23, 28.11, 28.08, 28.03. **MADLI-TOF-MS** (m/z) [M+K]^+^ calcd for C_28_H_40_O_8_K 543.2743, found 543.4310.


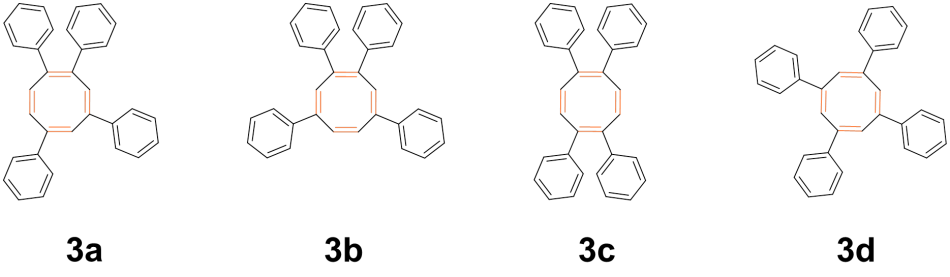


The title compound (**3a**-**3d**) was prepared by phenylacetylene, and the yield of isomer mixture was 56.6% after ball milling for 9h. (light yellow powder, 100:1 petroleum ether/ ethyl acetate). **^1^H NMR** (400 MHz, CDCl_3_, δ) 7.77, 7.75, 7.71, 7.69, 7.58, 7.57, 7.55, 7.53, 7.51, 7.49, 7.48, 7.46, 7.44, 7.42, 7.41, 7.40, 7.37, 7.35, 7.34, 7.29, 7.28, 7.24, 7.22, 7.20, 7.18, 7.00, 6.98, 6.91, 6.90, 6.80, 6.71, 6.71. **^13^C NMR** (101 MHz, CDCl_3_, δ) 144.60, 143.82, 143.44, 142.91, 142.79, 142.22, 141.57, 140.27, 139.95, 139.81, 139.78, 139.68, 130.34, 130.13, 130.11, 128.72, 128.68, 128.54, 128.52, 128.45, 128.37, 128.13, 128.00, 127.96, 127.84, 127.80, 127.55, 127.51, 127.34, 127.13, 127.05, 126.79, 126.74, 126.71, 126.38, 126.27. **MADLI-TOF-MS (m/z)** [M+H]^+^ calcd for C_32_H_25_ 409.1878, found 409.3440.


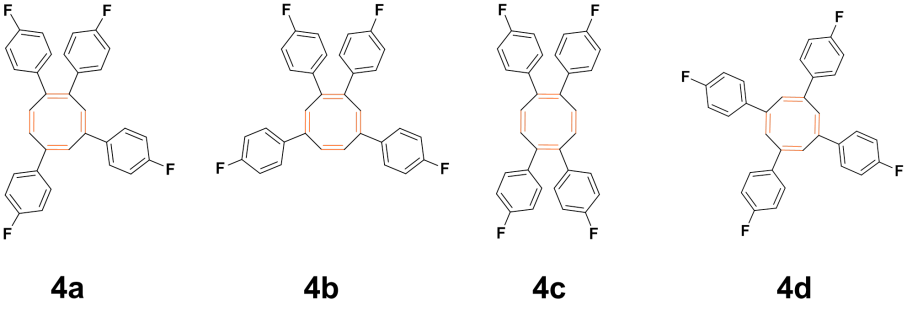


The title compound (**4a**-**4d**) was prepared by 4-fluorophenylacetylene, and the yield of isomer mixture was 25.5% after ball milling for 9h. (light yellow powder, 100:1 petroleum ether/ ethyl acetate). **^1^H NMR** (400 MHz, CDCl_3_, δ) 7.61, 7.59, 7.59, 7.57, 7.55, 7.53, 7.51, 7.44, 7.42, 7.41, 7.38, 7.37, 7.36, 7.35, 7.34, 7.32, 7.32, 7.30, 7.13, 7.11, 7.09, 7.06, 7.04, 7.02, 6.92, 6.91, 6.89, 6.80, 6.78, 6.73, 6.59, 6.54, 1.56. **^13^C NMR** (101 MHz, CDCl_3_, δ) 164.10, 163.96, 163.72, 163.55, 161.63, 161.50, 161.26, 161.09, 143.72, 142.36, 142.04, 141.66, 141.25, 140.46, 136.14, 136.11, 135.86, 135.83, 135.66, 135.63, 135.47, 135.44, 135.34, 135.31, 130.02, 129.93, 129.57, 128.78, 128.70, 128.41, 128.34, 128.27, 128.19, 127.93, 127.84, 127.83, 127.75, 127.04, 115.85, 115.78, 115.74, 115.63, 115.58, 115.53, 115.43, 115.41, 115.36. **MADLI-TOF-MS** (m/z) [M+H]^+^ calcd for C_32_H_21_F_4_ 481.1501, found 481.3090.

**Analysis of the distribution of the loss of mechanical energy in a reaction**

Based on the previous literature on the discrete-element-method (DEM) simulations of the planetary ball mills, we can give better insight into the mechanisms inside the grinding chamber like the motion, the collisions of balls and balls with the wall as well as the energy input. And several different variables influence the output energy of ball milling, such as the revolution speed, milling time and filling mass of milling balls. In addition, the filling balls indicate a very regular motion pattern and provide the kinetic energy during milling progress. For classical mechanics, the kinetic energy of an object of mass *m* moving at a speed *v* is 1/2 *mv*^2^, so the mechanical energy of planetary ball mills could be simplified to the following equation [[2](#_ENREF_2)]:

$E_{total}=\frac{1}{2}\times K_{a}m_{b}W_{P}^{2}R_{P}^{2}$ (1)

where $K_{a}$ is a constant describing the property of collision, $m_{b}$ is the mass of ball, $W_{p}$ and $R_{p}$ are corresponding to the rotational speed and radius of ball, respectively. The constant speeds and number of balls could provide the similar mechanical energy for the system in the same planetary ball mill.

For the metal catalytic system in this paper, the mechanical energy E*_total_* will be applied to the activation and pulverization of metal catalyst, the diffusion and mass transport of reaction system. Due to the presence of strong metallic bonds and dense crystal structure, solid metals typically exhibit high modulus and hardness (equation 3) [[3](#_ENREF_3)]. However, the liquid gallium exhibits fluid properties like water at room temperature due to its long-range disordered amorphous structure, with a Young’s modulus approaching zero [[4](#_ENREF_4)]. Hence, liquid metal requires less energy for pulverization or deformation compared to solid metal (equation 4). The equations are as follows:

$E_{Y}\propto\frac{E_{b}}{r_{e}^{3}}$ (2)

$W\geq\frac{1}{2}E_{Y}\varepsilon^{2}$ (3)

where *E_Y_* is the Young’s modulus, *E_b_* is the atomic bond energy, *r_e_* is the bond length, W represents the energy required to crush the metal, $\varepsilon$ is the strain of metal.

**Finite element simulation of the mass transfer process in solid-liquid metal catalysts**

In a system of reactants with constant concentration, a micrometer-scale hemispherical catalyst model was established and considered this model as an independent catalytic unit (As shown in Figure 4B). We assume that this catalyst system is an infinitely sustainable first-order irreversible reaction process and the reactants are uniformly distributed around the catalyst. For traditional solid catalysts, the catalytic reaction process only occurs at the interface region of catalysts, reactive mass transfer can be expressed by the following system of equations:

$R_{eq}:K_{eq}=\frac{\prod_{j\epsilon products} \left( c_{j}/c_{a0} \right)^{v_{j}}}{\prod_{j\epsilon reactants} \left( c_{j}/c_{a0} \right)^{{-v}_{j}}}$ (4)

$\nabla\cdot\mathbf{J}_{j}+\mathbf{u}\cdot\nabla c_{j}=v_{j}R_{eq}$ (5)

where the $v_{j}$ is stoichiometric number of reaction, $R_{\mathrm{eq}}$ and $K_{\mathrm{eq}}$ are the reaction rate and equilibrium constant respectively. $v_{j}R_{\mathrm{eq}}$ denotes the localized concentration changes caused by reaction. $\nabla\cdot J_{j}$ and $u\cdot\nabla c_{j}$ are the diffusion concentration at the catalyst interface and the concentration changes caused by external action respectively.

Different from solid catalysts, liquid metals exhibit convective motion within the metal bulk phase. Therefore, the catalytic systems need to account for just diffusion mass transfer between reactants and catalysts, but also convective mass transfer. This can be described by the following set of equations, the system of equations can describe the change in convection-diffusion concentration during the reaction can be described by the following system of equations:

$\nabla\cdot\boldsymbol{J}_{j}+\mathbf{u}\cdot\nabla c_{j}=R_{j}$ (6)

$\boldsymbol{J}_{j}=-D_{j}\nabla c_{j}$ (7)

And the process of reactants entering the interior of catalyst and diffusing to the exterior can be denoted by the system of equations:

$-\mathbf{n}\cdot\mathbf{J}_{j,u}=\frac{D_{s,j}}{d_{s}}\left( c_{j,d}-c_{j,u} \right)$ (8)

$-\mathbf{n}\cdot\mathbf{J}_{j,d}=\frac{D_{s,j}}{d_{s}}\left( c_{j,u}-c_{j,d} \right)$ (9)

where the $\boldsymbol{J}_{j}$ is the mass transfer flux, $D_{j}$ is the diffusion coefficient, $\mathbf{u}$ represnets the velocity of fluid flow inside the metal, $R_{j}$ is reaction rate, $\nabla\cdot\boldsymbol{J}_{j}$ is the spatial gradient of mass transfer flux, which describes the concentration variations at different points in the fluid arising from substance diffusion. $\mathbf{u}\cdot\nabla c_{j}$ denotes the concentration variations at different locations caused by the flow of liquid metal. $D_{s,j}$ represents the diffusion conefficient in the potential barrier. $d_{s}$ is the thickness of the potential barrier layer.$c_{j,d}-c_{j,u}$ represents the concentration difference to either side of the potential barrier.

**Characterization details of active Mg in L-GaMg**

Synthesis procedures: 6 magnesium particles (Ø 3 mm), 0.5g liquid gallium, 4 ZrO_2_ balls (Ø 10 mm), 14 ZrO_2_ balls (Ø 5 mm) and 14 ZrO_2_ balls (Ø 2 mm) were ball-milled in a 25 ml jar under an inert atmosphere for 30 minutes (at speed of 270 rad/min ).

SEM: the obtained L-GaMg was promptly characterized by SEM, followed by video recording of a selected area for observation (30-60min).

XPS: the obtained L-GaMg was rapidly cooled to solidify and stored at 5℃ to prevent liquefaction. Then the XPS characterization was immediately performed after a 5-minute etching pretreatment to remove the surface oxide layer.

It is crucial that all procedures be performed under an inert atmosphere throughout.

**Characterization details of active Ni in L-GaNi**

Synthesis procedures: 4 nickel metal balls (Ø 10 mm), 0.5g liquid gallium, 14 ZrO_2_ balls (Ø 5 mm) and 14 ZrO_2_ balls (Ø 2 mm) were ball-milled in a 25 ml jar under an inert atmosphere for 2 h (at speed of 270 rad/min ). The nickel balls should be ground to remove the oxide film before being used.

XPS: the obtained L-GaNi was rapidly cooled to solidify and stored at 5℃ to prevent liquefaction. Then the XPS characterization was immediately performed after a 5-minute etching pretreatment to remove the surface oxide layer.

It is crucial that all procedures be performed under an inert atmosphere throughout.

**Supplementary Figures**


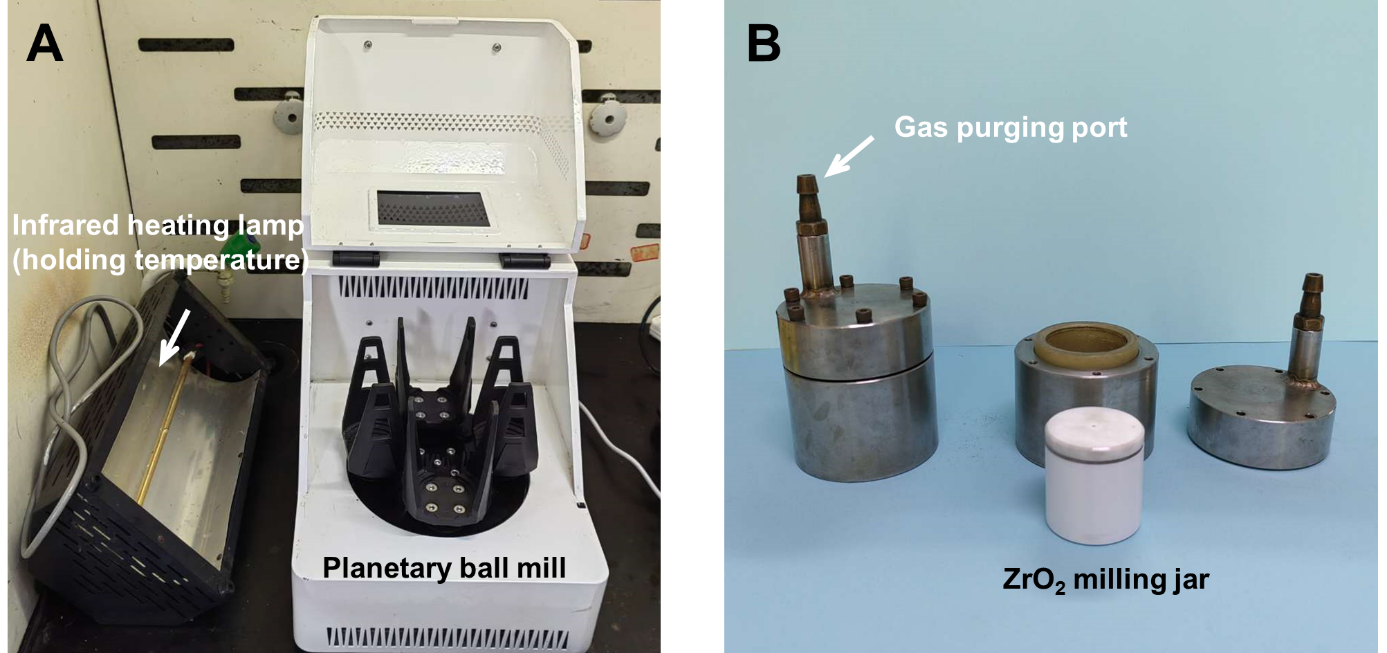


**Fig. S2.** Optical photograph of the reaction device. (A) Planetary ball mill. (B) Milling jar with gas outlet/inlet valves.


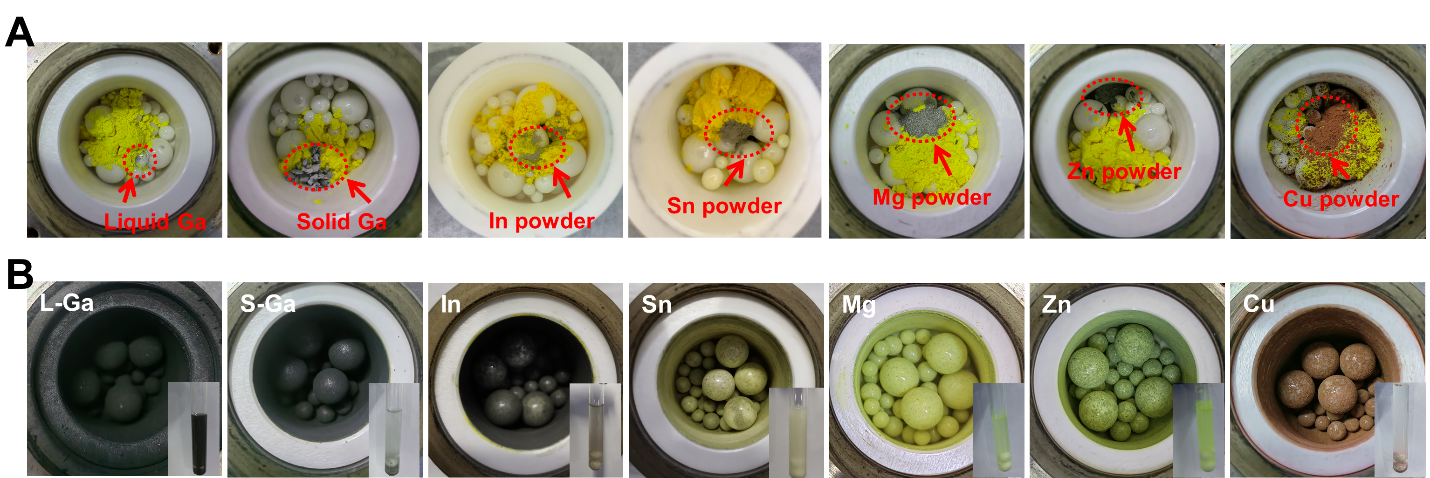


**Fig. S3.** Optical photographs of TBPB monomer before and after reaction. (A) Digital images of different metals before ball milling. (B) Digital images of different metals after ball milling 1 h (the inside image shows the product after hydrochloric acid washing).


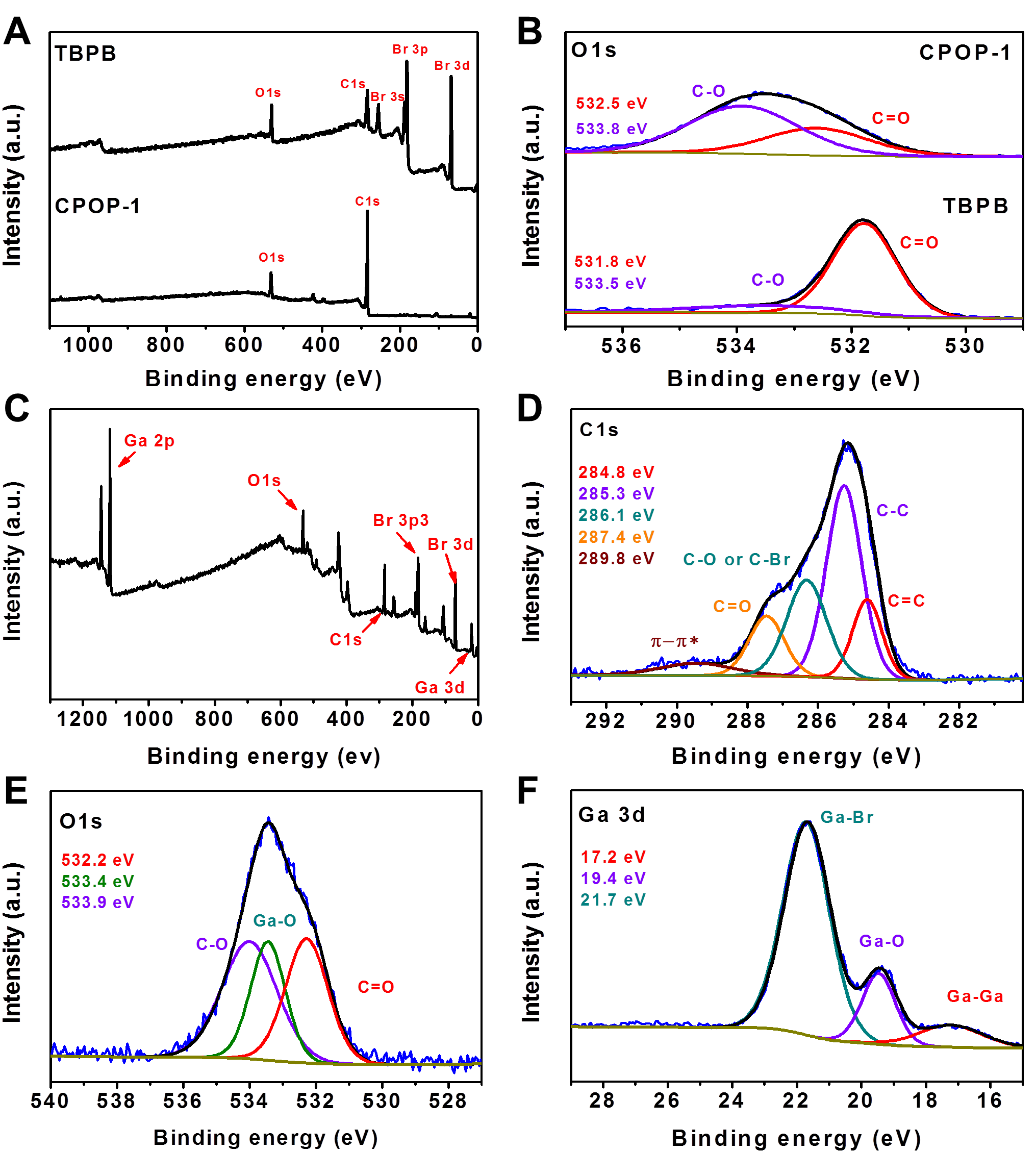


**Fig. S4.** XPS characterization of TBPB monomer, CPOP-1 product and reaction mixture. (A) and (B) XPS survey spectrum and O1s spectrum of the TBPB monomer and CPOP-1 product. (C-F) XPS survey spectrum and C1s, O1s, Ga3d spectrum of the reaction mixture of TBPB monomer and liquid gallium (ball milling for 1 h).


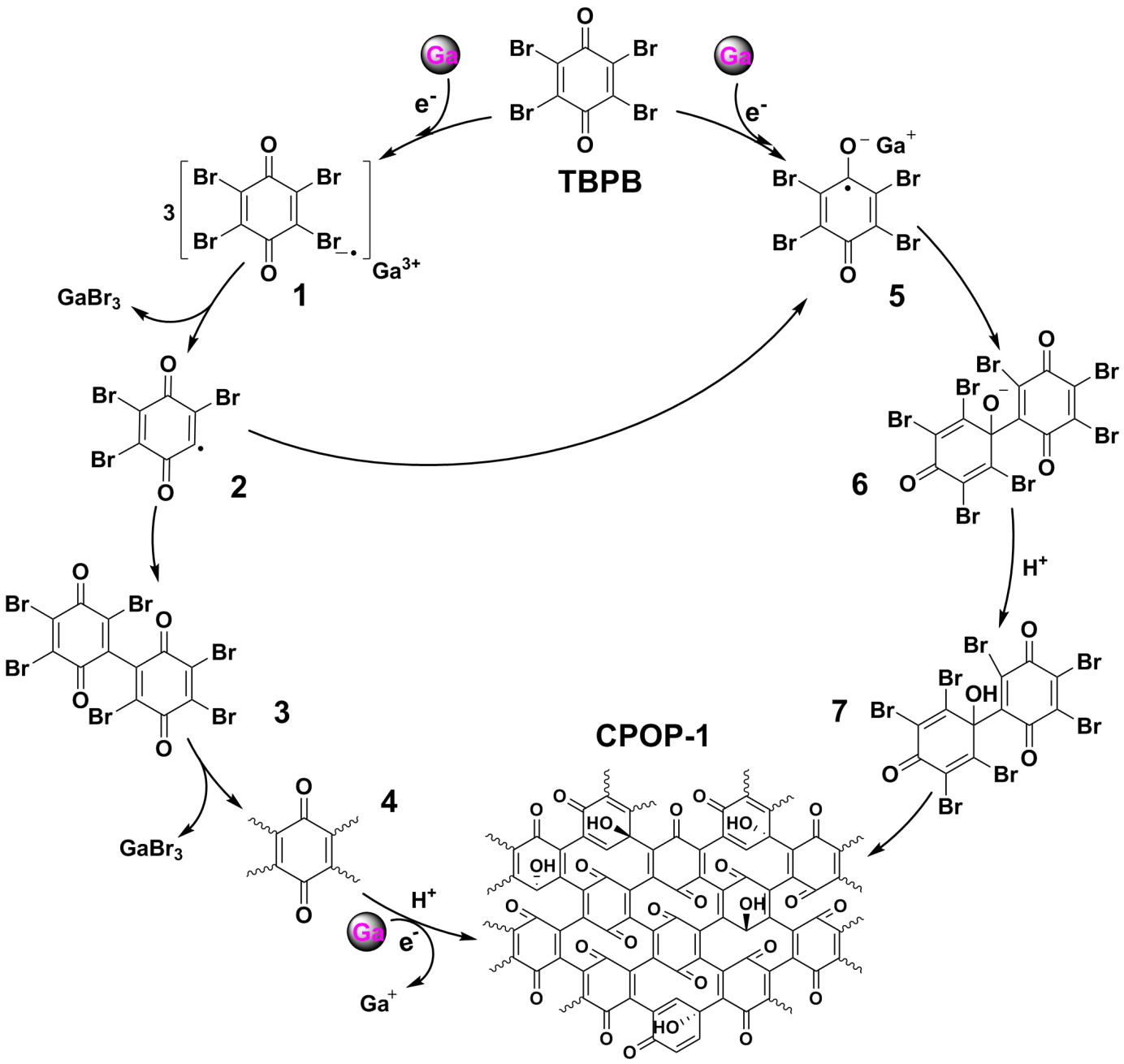


**Fig. S5.** Proposed reaction mechanism for the construction of CPOP-1 from TBPB monomer via single-electron transfer pathway.


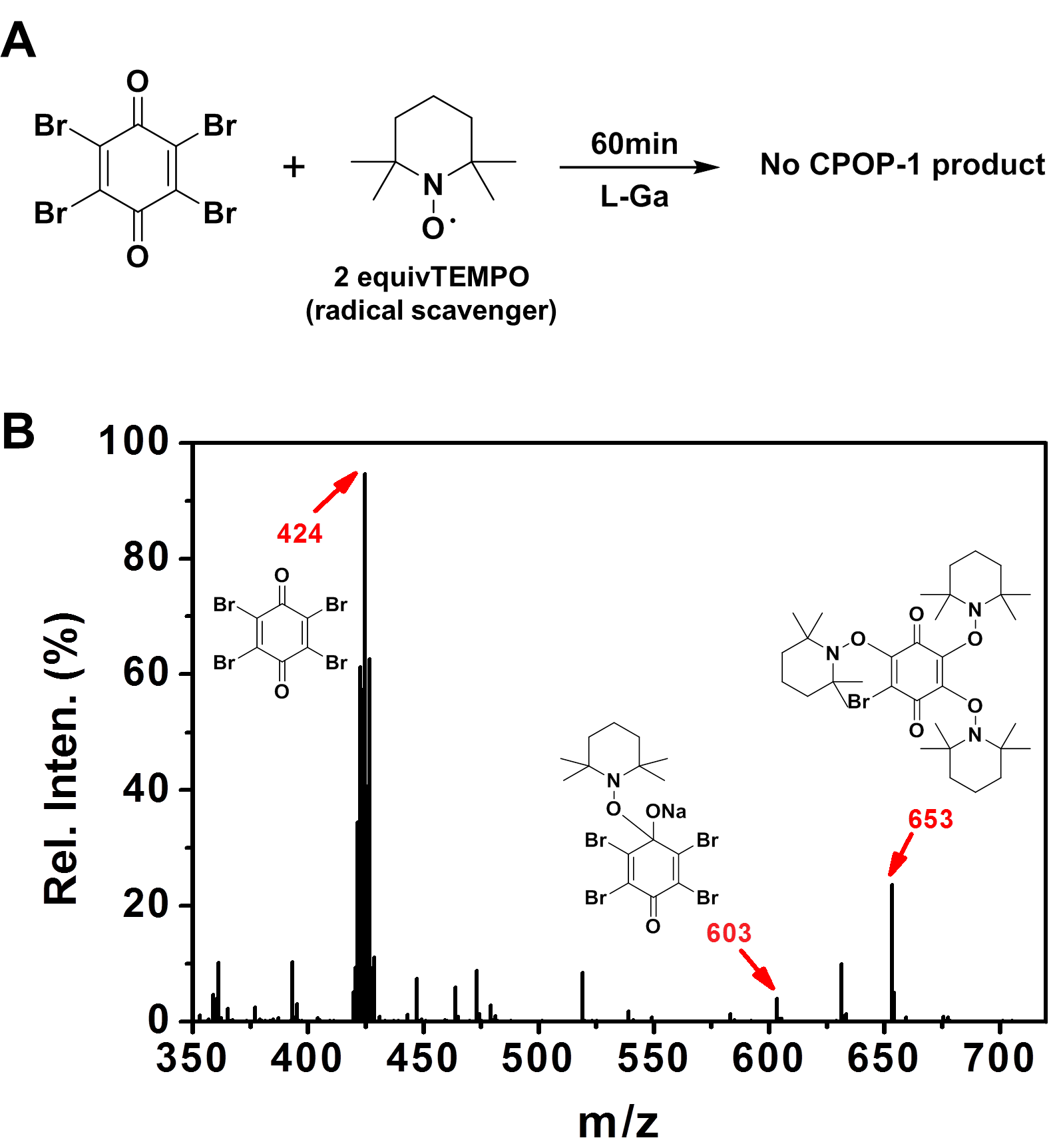


**Fig. S6.** Free radicals capture validation experiment. (A) The reaction equation of TBPB and TEMPO. (B) The HRMS spectrum of TBPB, liquid gallium and TEMPO ball milling for 1 h.


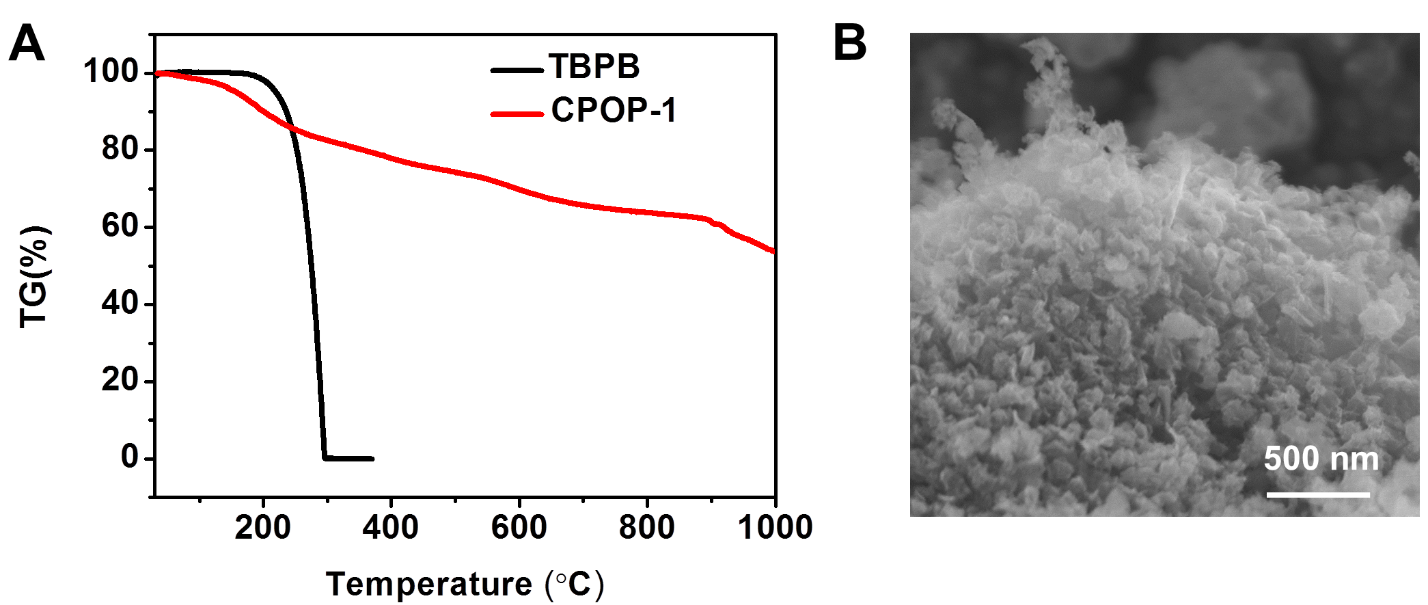


**Fig. S7.** Characterization of CPOP-1 product ball milling for 1 h. (A) TG curve of TBPB monomer and CPOP-1 product. (B) SEM image of CPOP-1 product after ball milling for 1h.


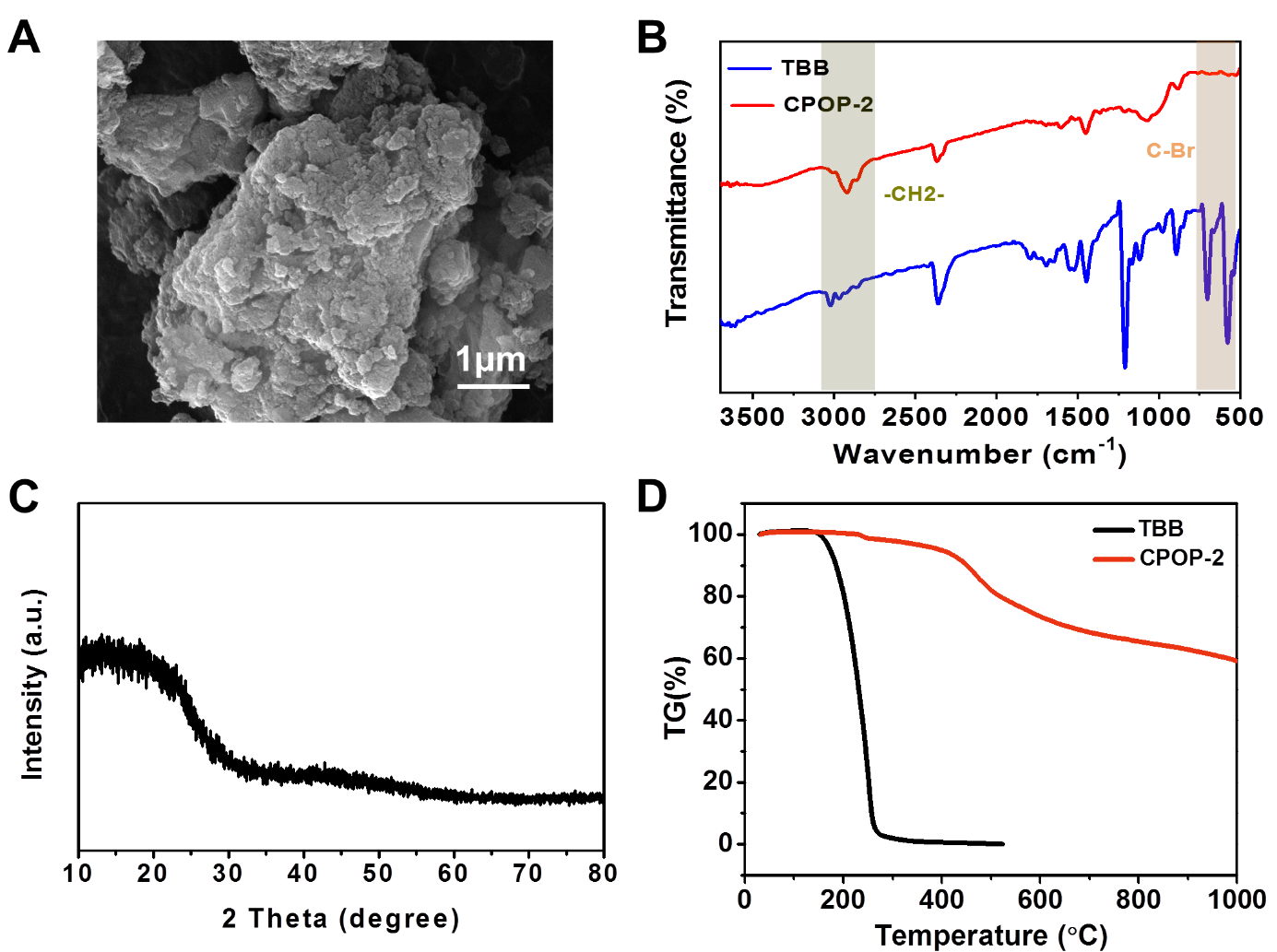


**Fig. S8.** Characterization of CPOP-2 product ball milling for 1 h. (A) SEM image. (B) FT-IR spectra of CPOP-2 product and TBB monomer. (C) XRD spectra. (D) TG spectra of CPOP-2 product and TBB monomer.


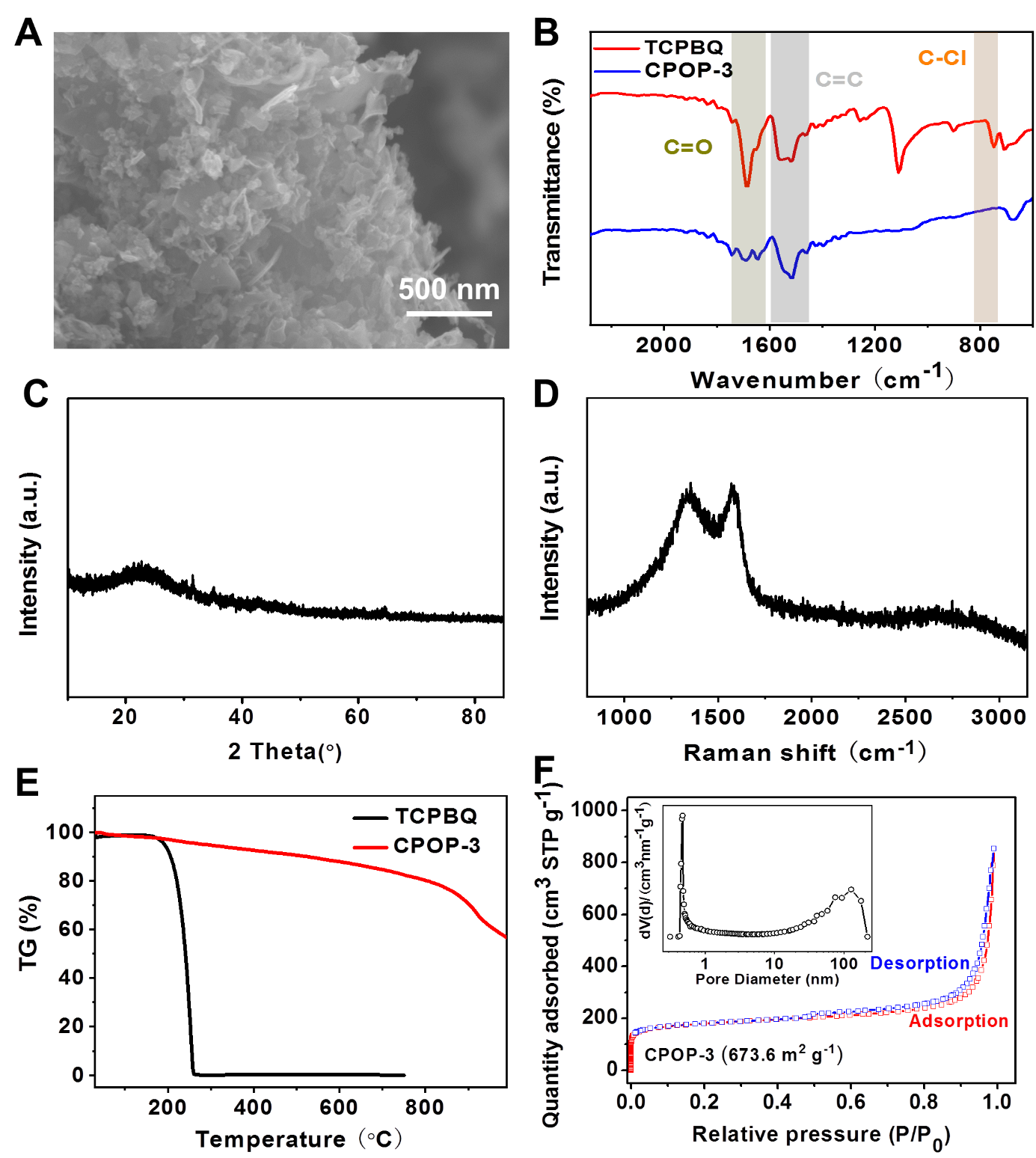


**Fig. S9.** Characterization of CPOP-3 product ball milling for 1 h. (A) SEM image of CPOP-3 product. (B) FT-IR spectra of CPOP-3 product and TCPBQ monomer. (C) XRD spectra of CPOP-3 product. (D) Raman spectra of CPOP-3 product. (E) TG spectra of CPOP-3 product and TCPBQ monomer. (F) Nitrogen adsorption desorption isotherms of CPOP-3 product at 77K (Pore size distribution in the inner chart).


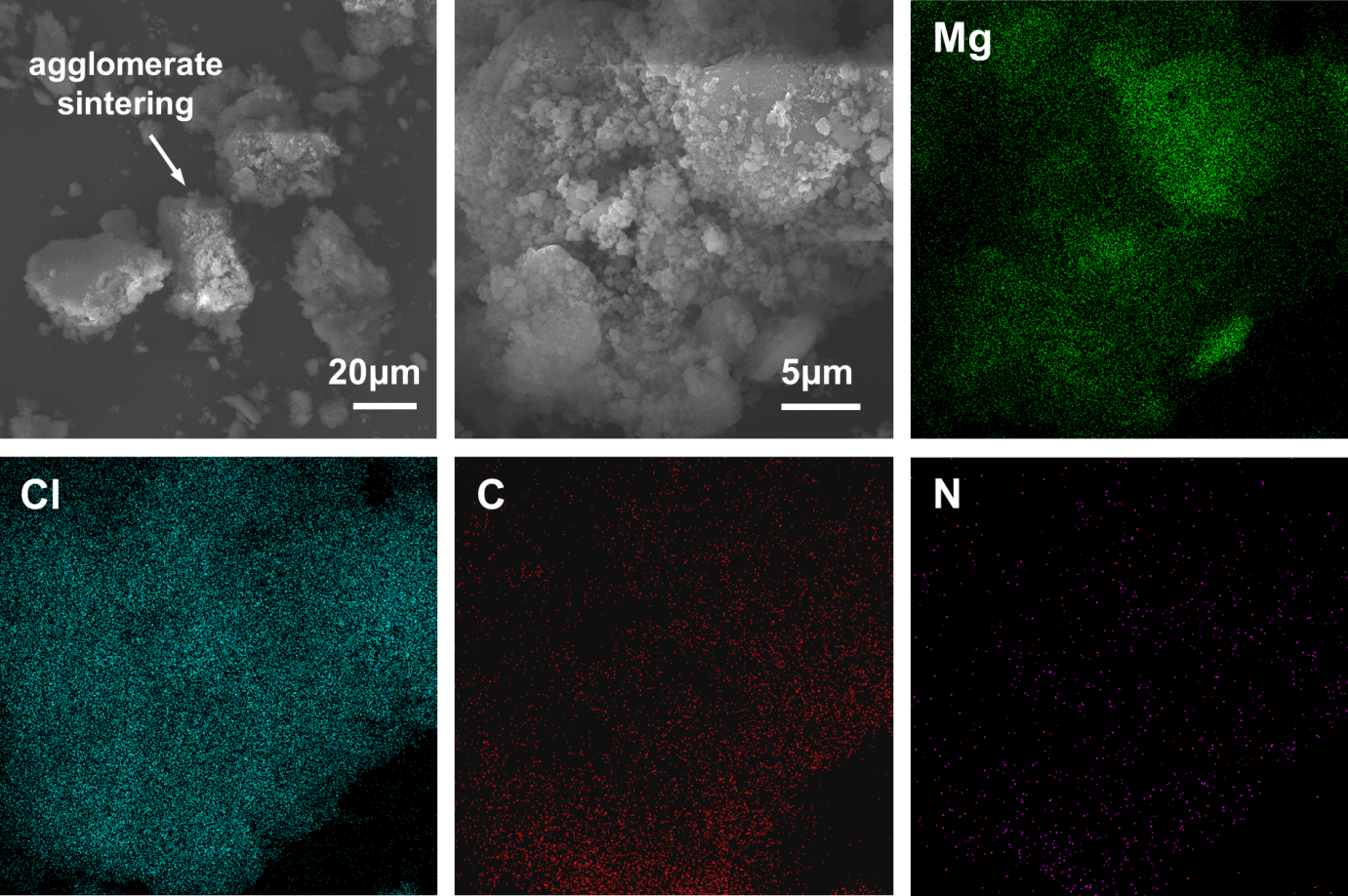


**Fig. S10.** SEM images and elemetal mappings of Mg, Cl, C, N of mixture (Mg powder and TCTPAN ball milling for 3 h).


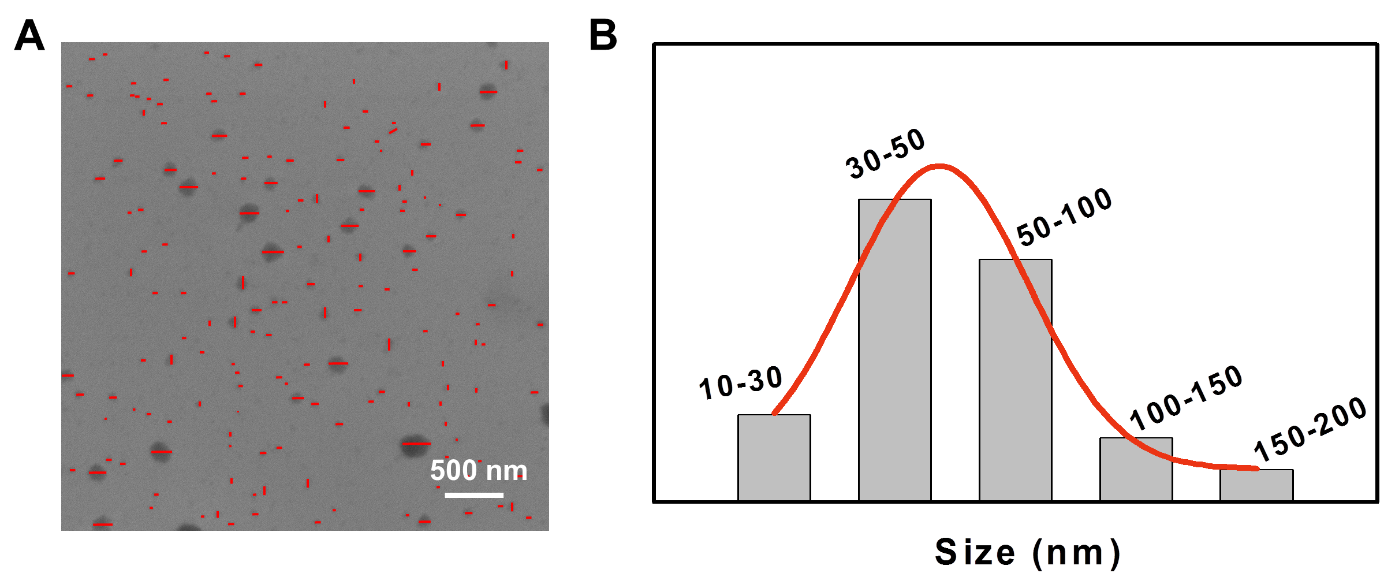


**Fig. S11.** (A) SEM images of active nano-Mg in liquid gallium (active Mg marked with red line). (B) Size distribution analysis of active nano-Mg.


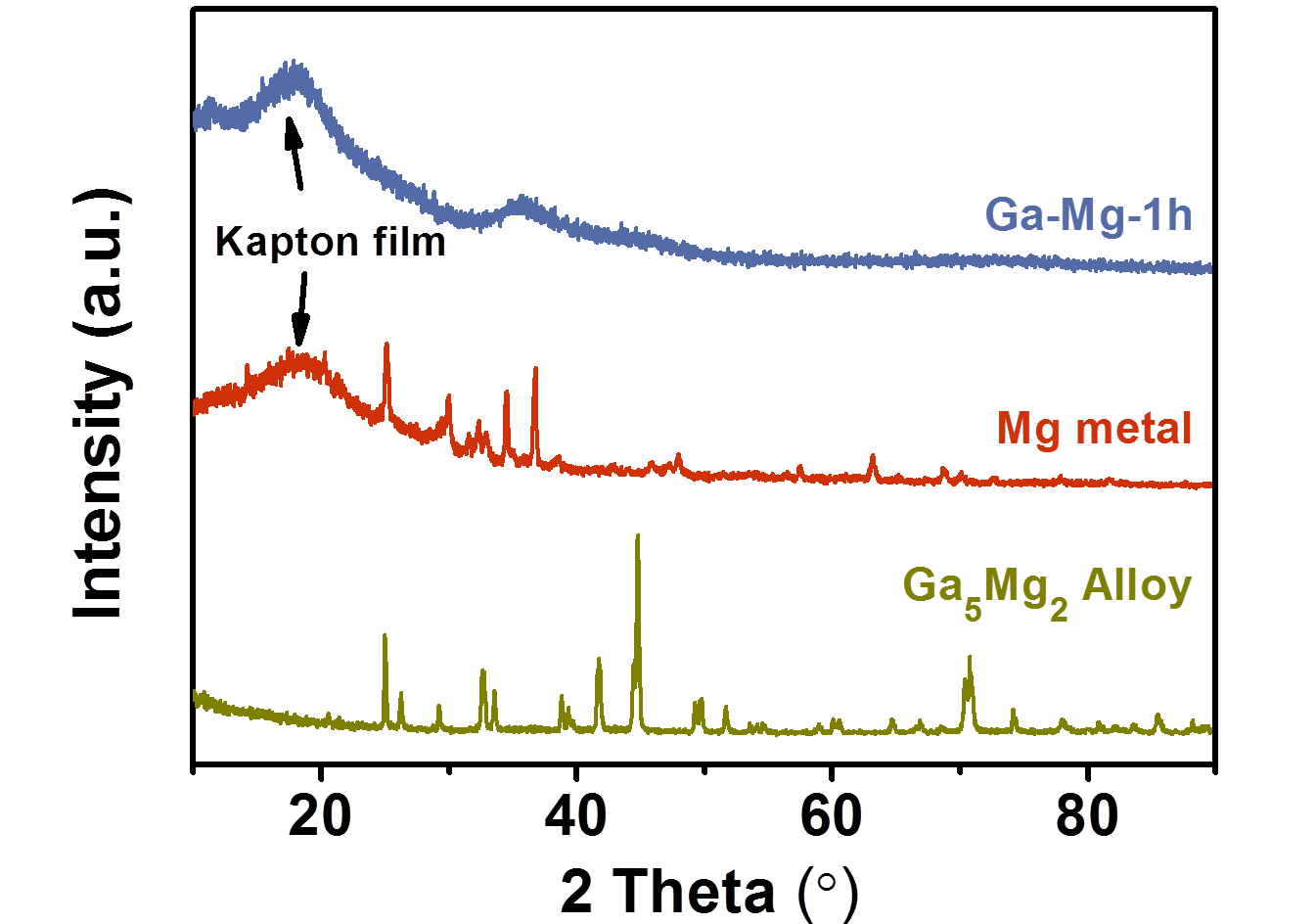


**Fig. S12.** XRD spectra of the Ga_5_Mg_2_ alloy, Mg powder and composite liquid Ga-Mg catalyst (ball milling for 1h).


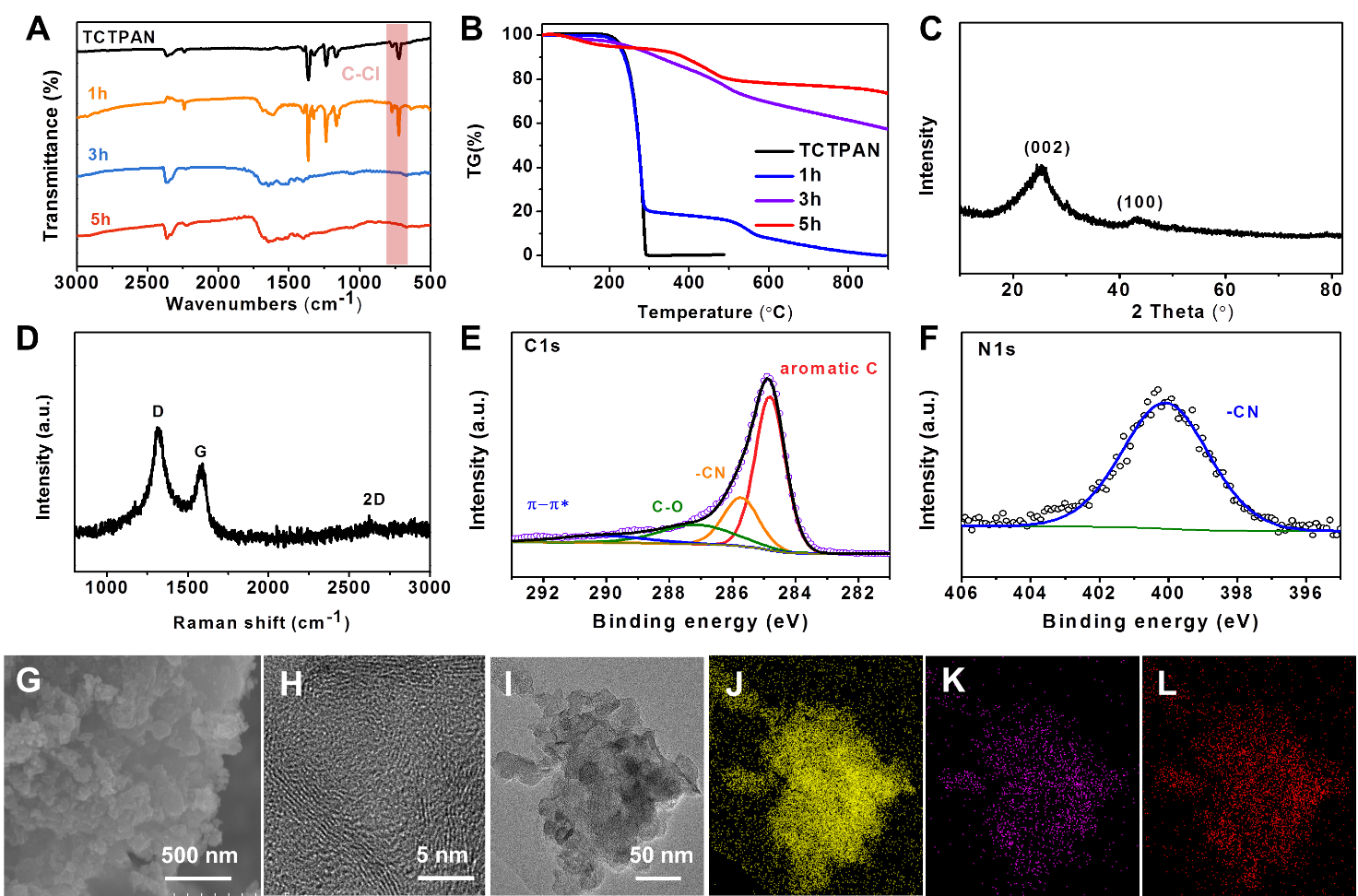


**Fig. S13.** Characterization of CPOP-4 product. (A) FT-IR spectra of CPOP-4 products with different reaction time. (B) TG spectra of CPOP-4 products with different reaction time. (C) XRD patterns of CPOP-4 product. (D) Raman spectra of CPOP-4 product. (E) and (F) C1s and N1s XPS spectra of CPOP-4 product. (G) SEM image of CPOP-4 product. (H-L) TEM images and EDS mapping of CPOP-4 product (j-l corresponds to the C, N, O elements respectively).


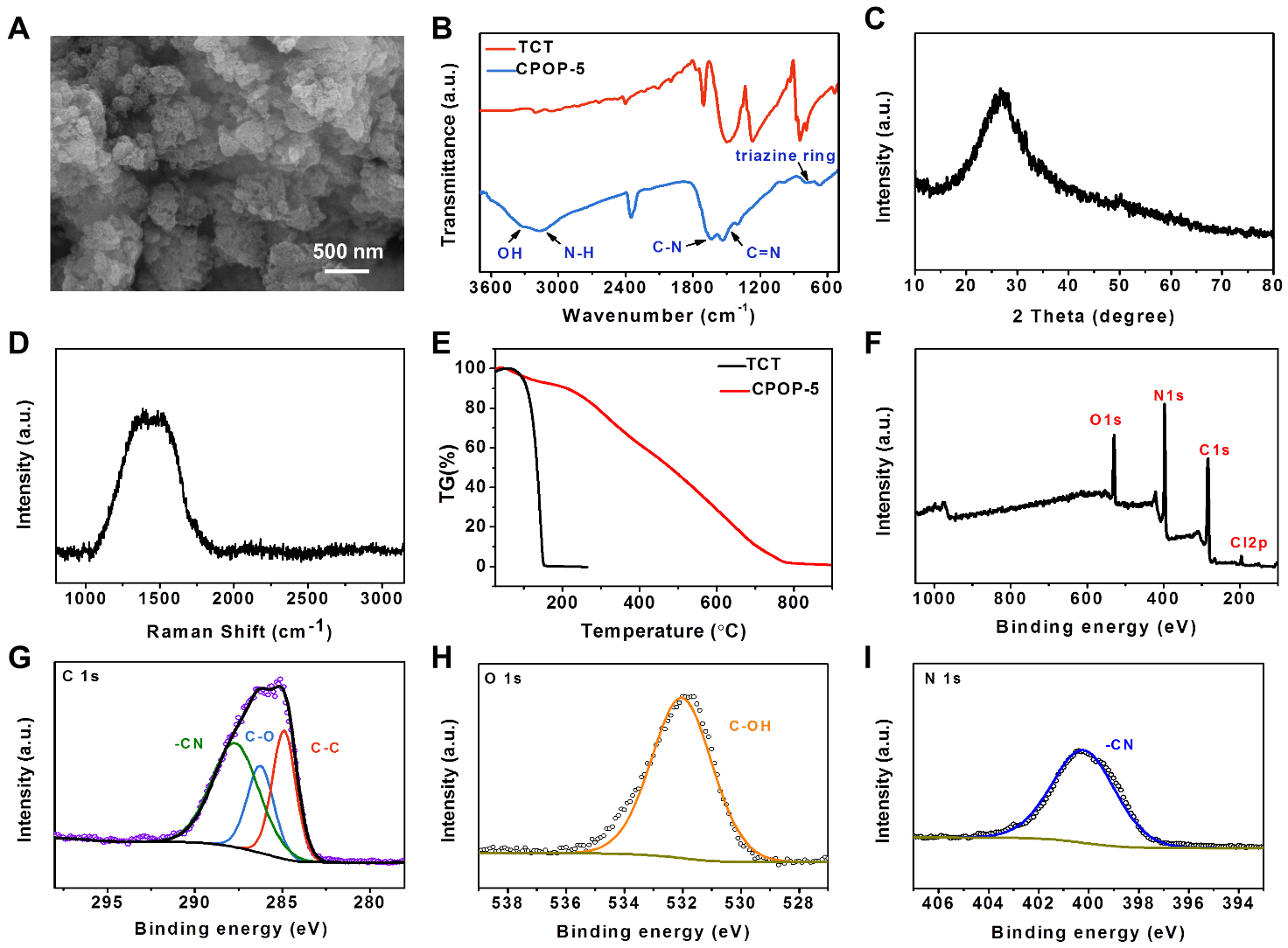


**Fig. S14.** Characterization of CPOP-5 product. (A) SEM image of CPOP-5 product. (B) FT-IR spectra of CPOP-5 product and TCT monomer. (C) XRD pattern of CPOP-5 product. (D) Raman spectra of CPOP-5 product. (E) TG spectra of CPOP-5 product and TCT monomer. (F-I) XPS survey spectrum, C1s, O1s and N1s spectra of CPOP-5 product.


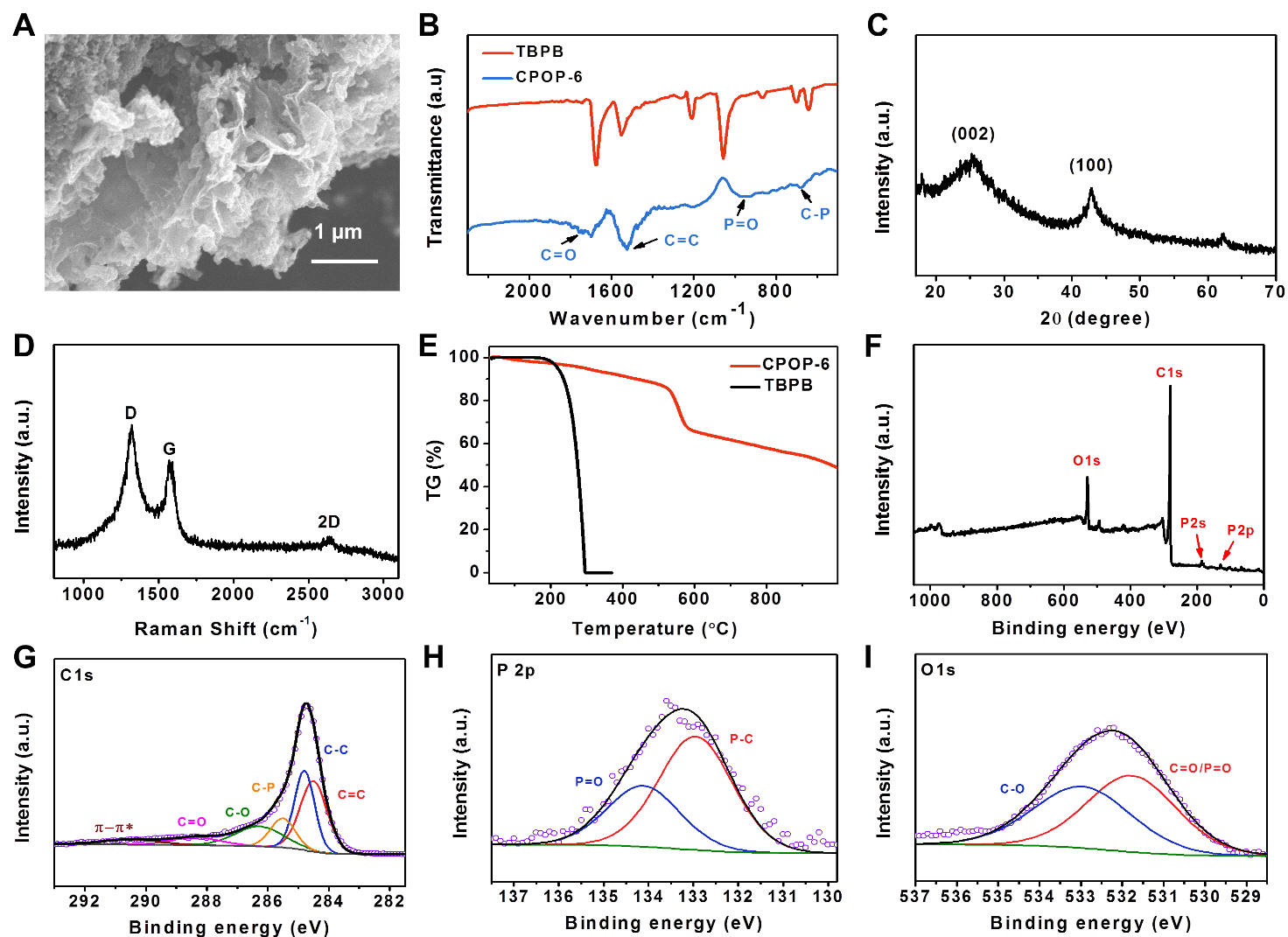


**Fig. S15**. Characterization of CPOP-6 product obtained by the coupling of TBPB and POC monomer. (A) SEM image of CPOP-6 product. (B) FT-IR spectra of CPOP-6 product and TBPB monomer. (C) XRD patterns of CPOP-6 product. (D) Raman spectra of CPOP-6 product. (E) TG spectra of CPOP-6 product and TBPB monomer. (F-I) XPS survey spectrum, C1s, P2p and O1s spectra of CPOP-6 product.


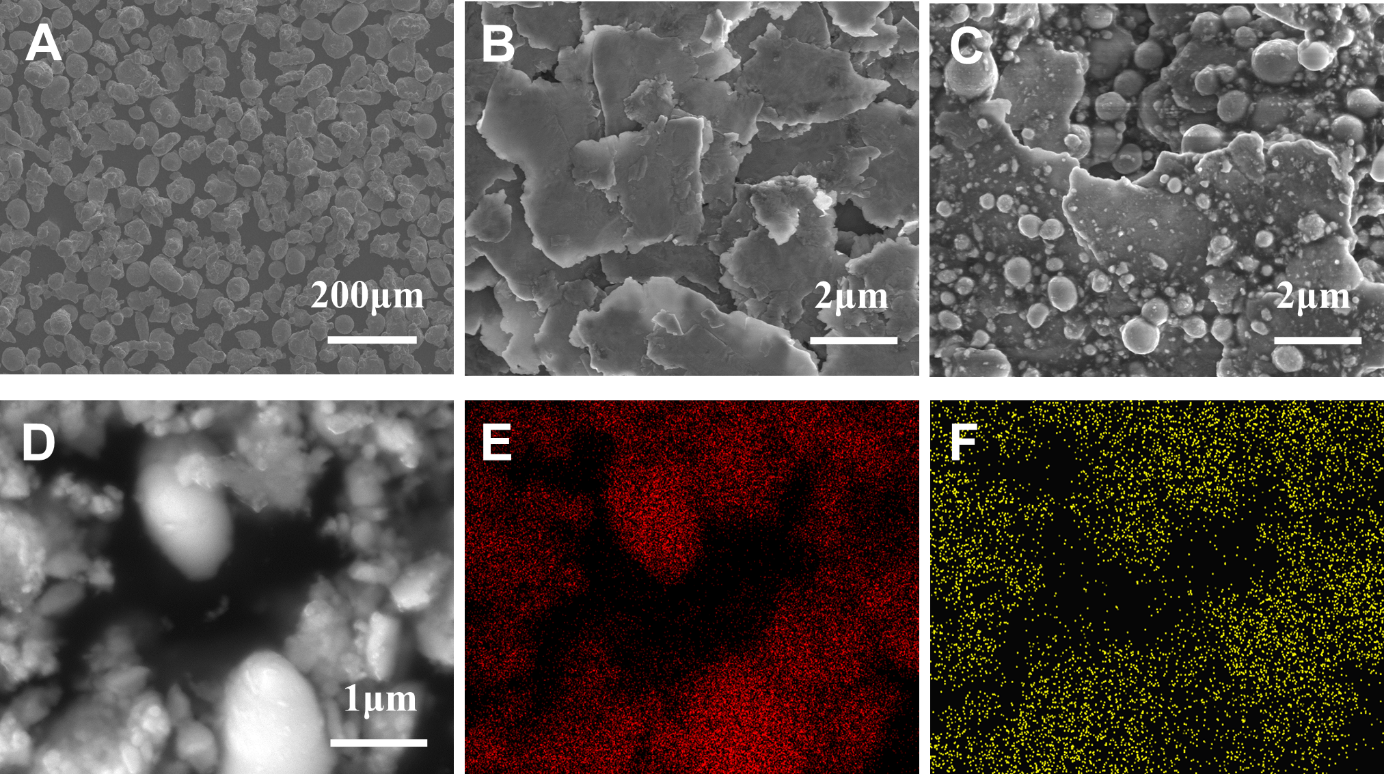


**Fig. S16.** SEM characterization of nickel catalyst and gallium-nickel catalyst after ball milling reaction. (A) SEM image of nickel powder before reaction. (B) SEM image of nickel powder and ethyl propiolate ball milling for 9h. (C) SEM image of nickel powder, liquid gallium and ethyl propiolate ball milling for 9h. (D-F) SEM image and EDS mappings of liquid Ga-Ni, e is the element of Ga, f is the element of Ni. (After nickel powder, liquid gallium and ethyl propiolate ball milling for 9h, ethyl acetate was added to disperse the mixture and the excess nickel was removed with a magnet to obtain liquid Ga-Ni).


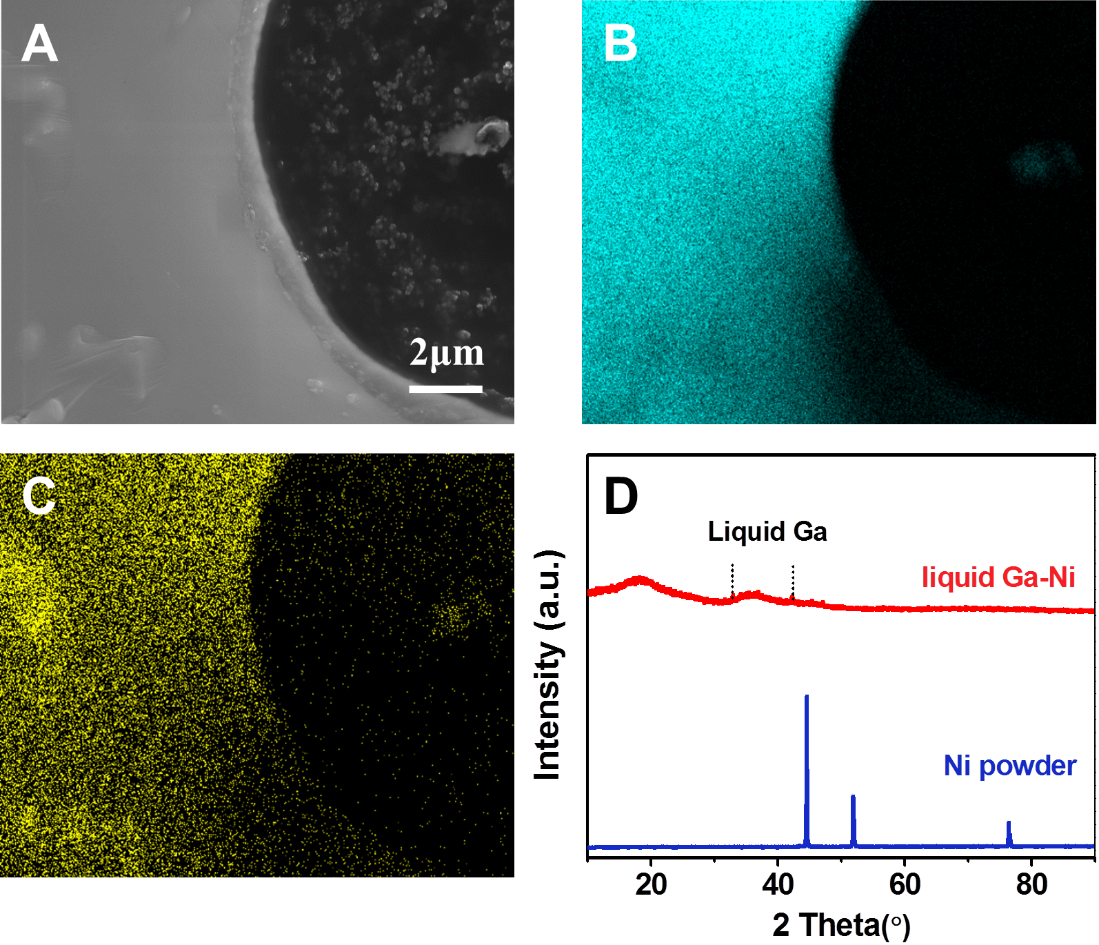


**Fig. S17.** Characterization of liquid Ga-Ni. (A) SEM image of liquid Ga-Ni after nickel particles and liquid gallium ball milling for 2h. (B) and (C) EDS mapping of liquid Ga-Ni, b is Ga element and c is Ni element. (D) XRD spectra of nickel particles and liquid Ga-Ni.

**
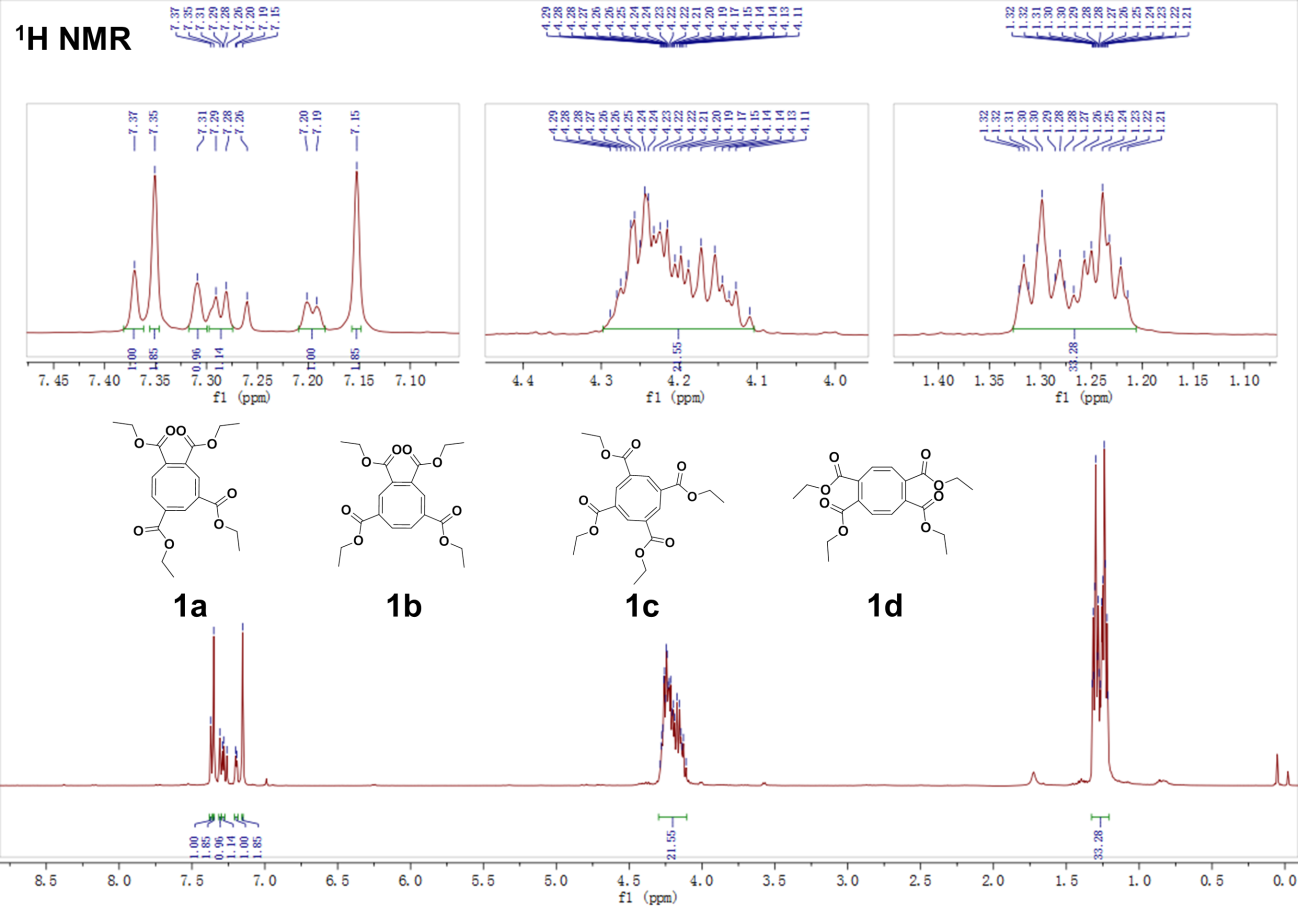
**

**
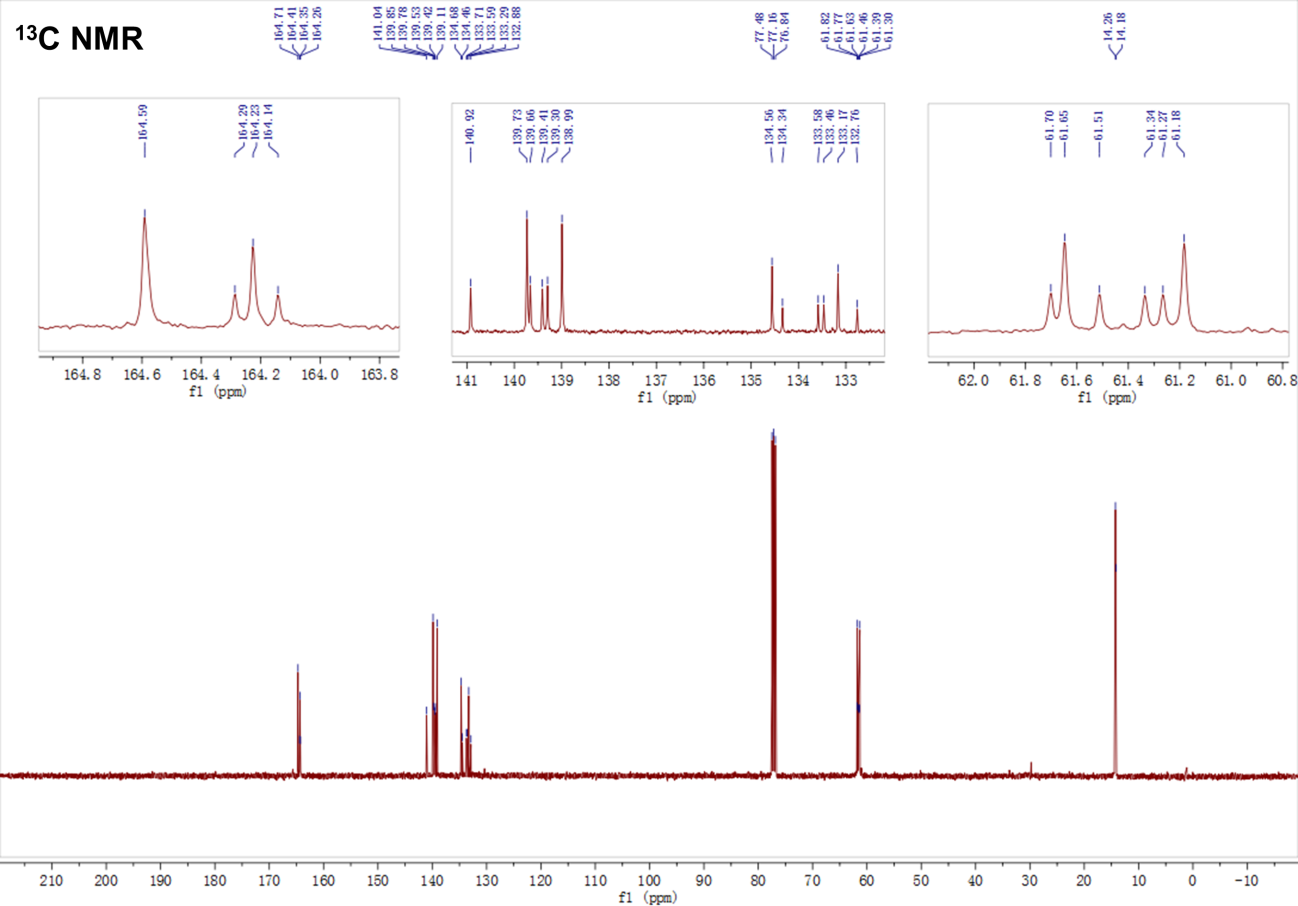
**

**Fig. S18.** ^1^H NMR and ^13^C NMR spectrum of cyclooctatetraene compounds synthesized by ethyl propiolate.

**
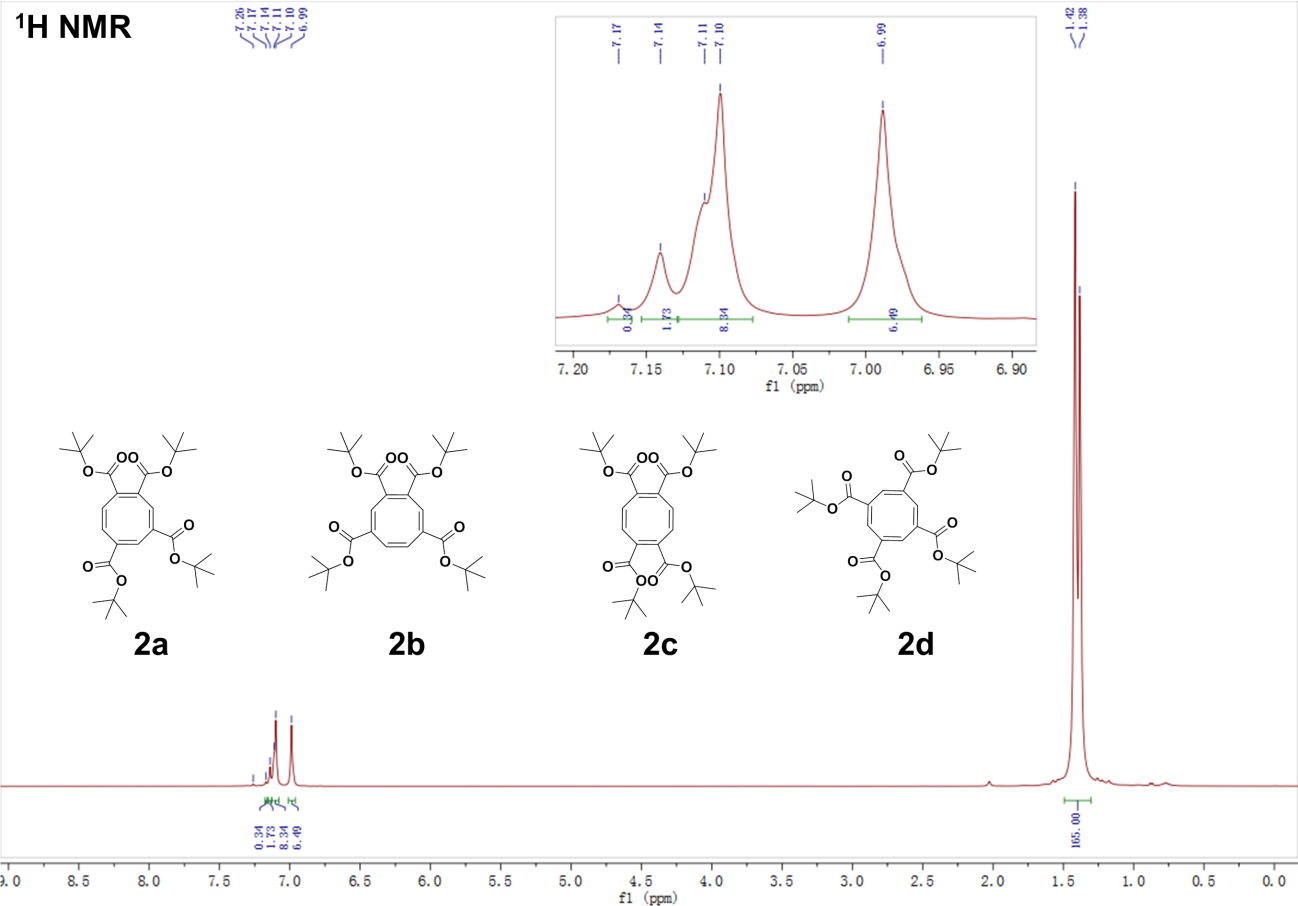
**

**
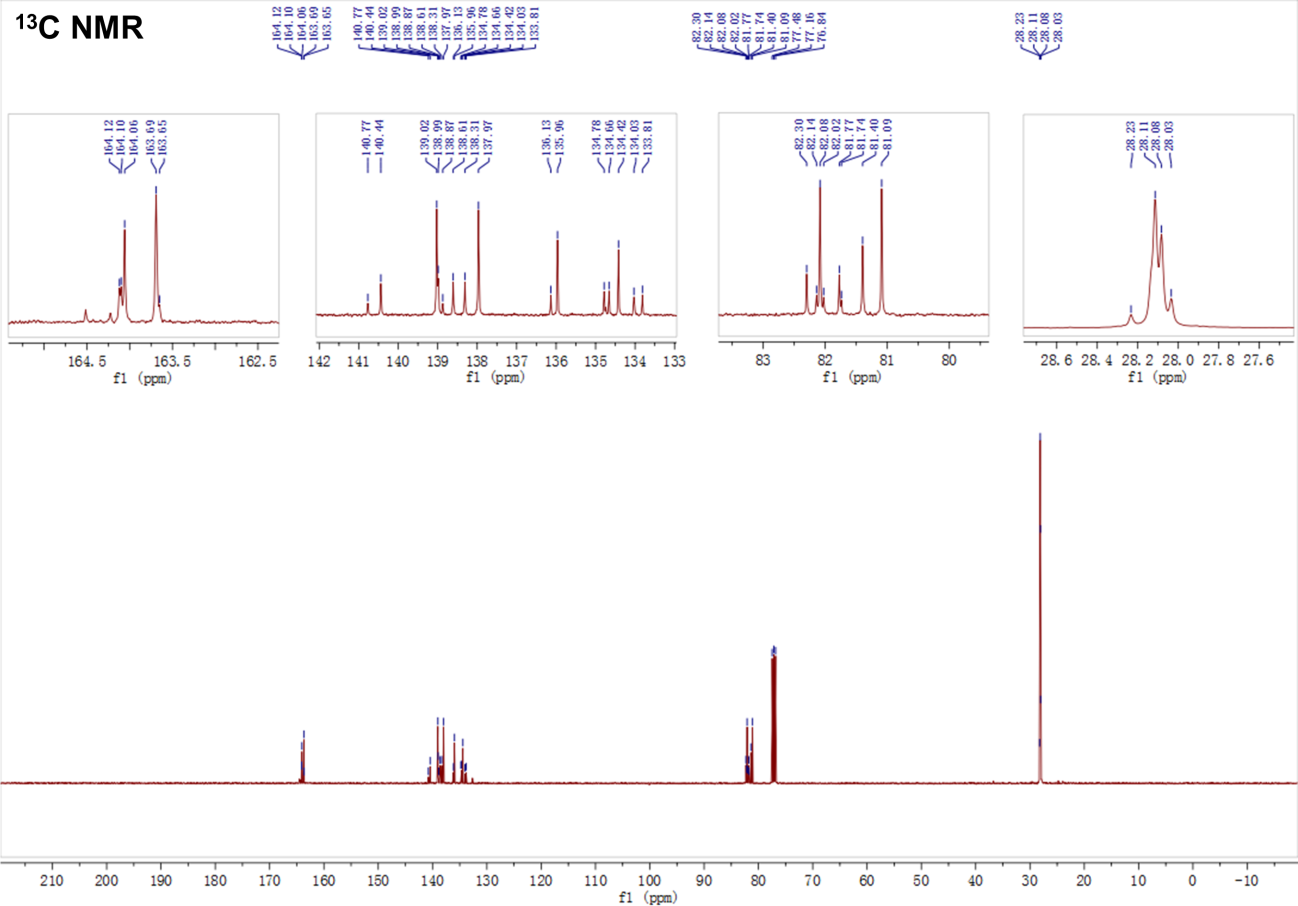
**

**Fig. S19.** ^1^H NMR and ^13^C NMR spectrum of cyclooctatetraene compounds synthesized by tert-butyl propiolate.

**
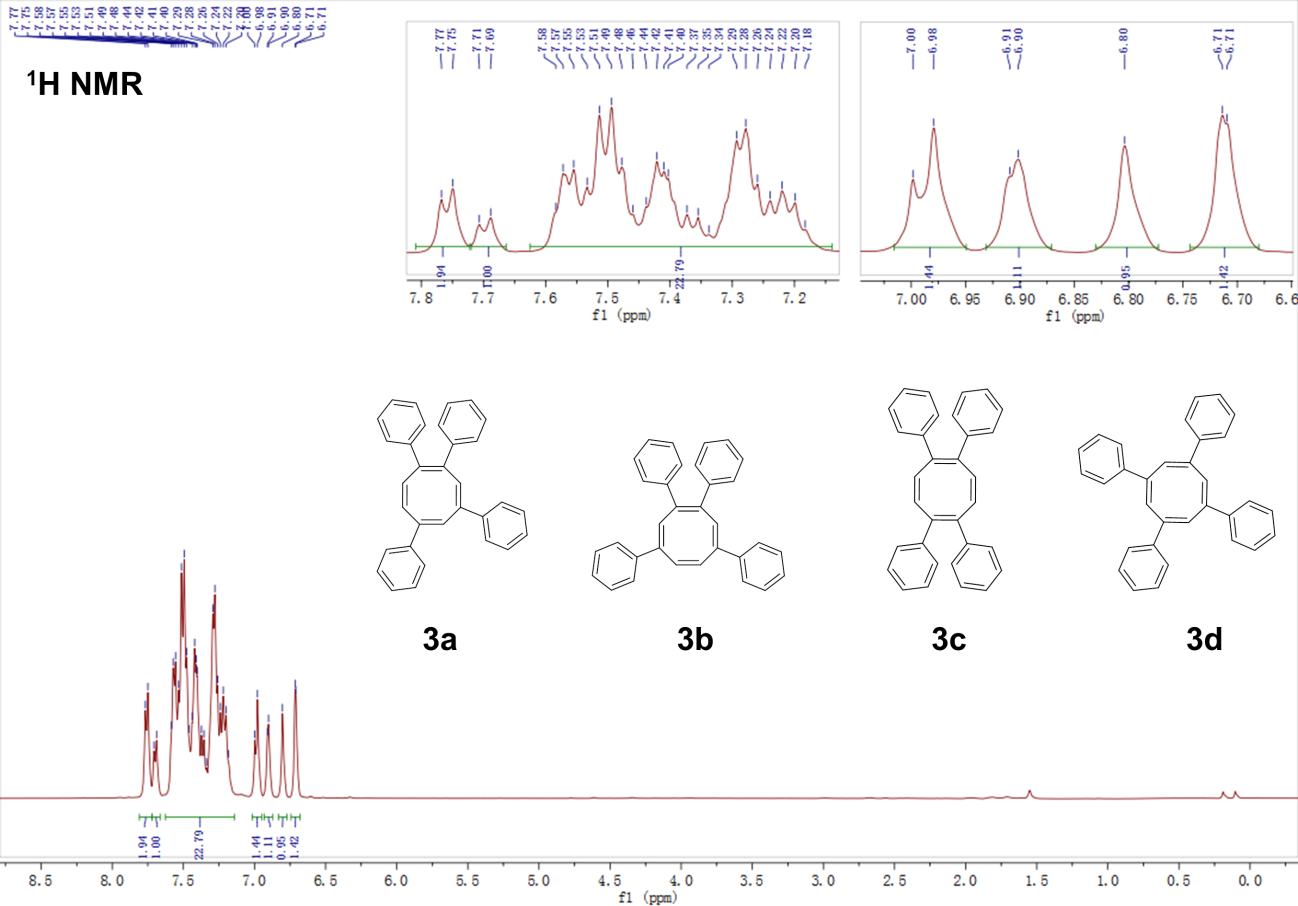
**

**
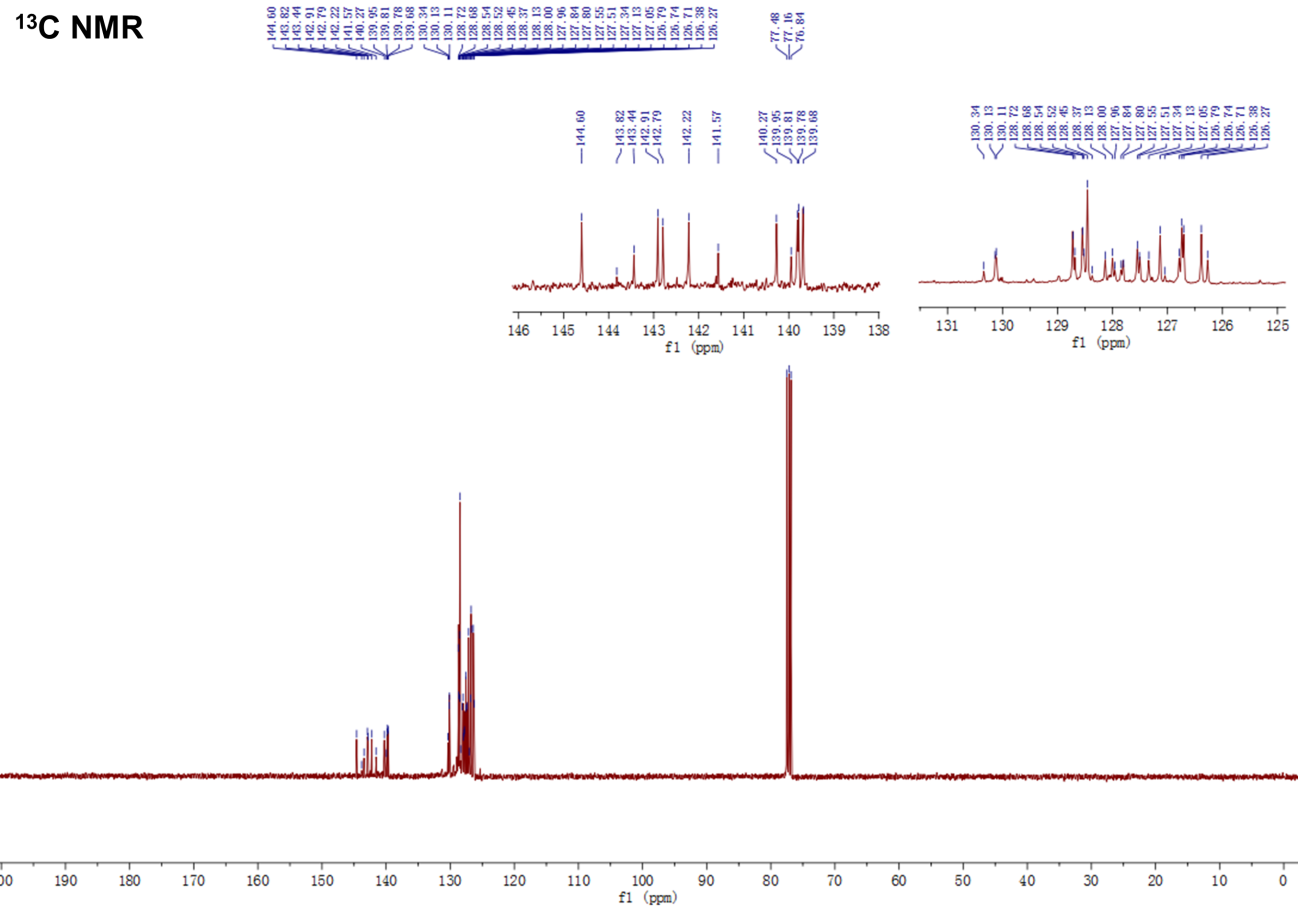
**

**Fig. S20.** ^1^H NMR and ^13^C NMR spectrum of cyclooctatetraene compounds synthesized by phenylacetylene.

**
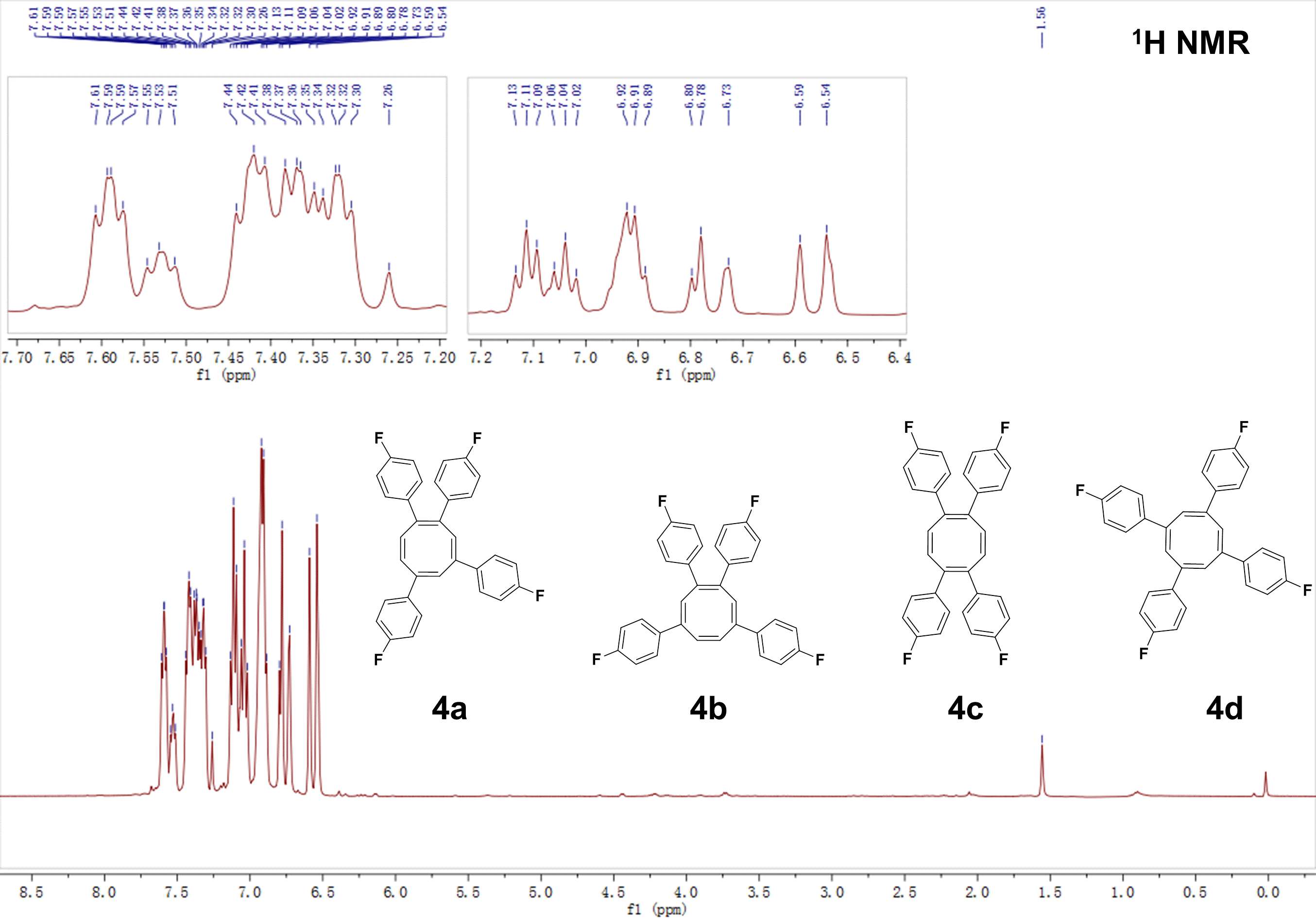
**

**
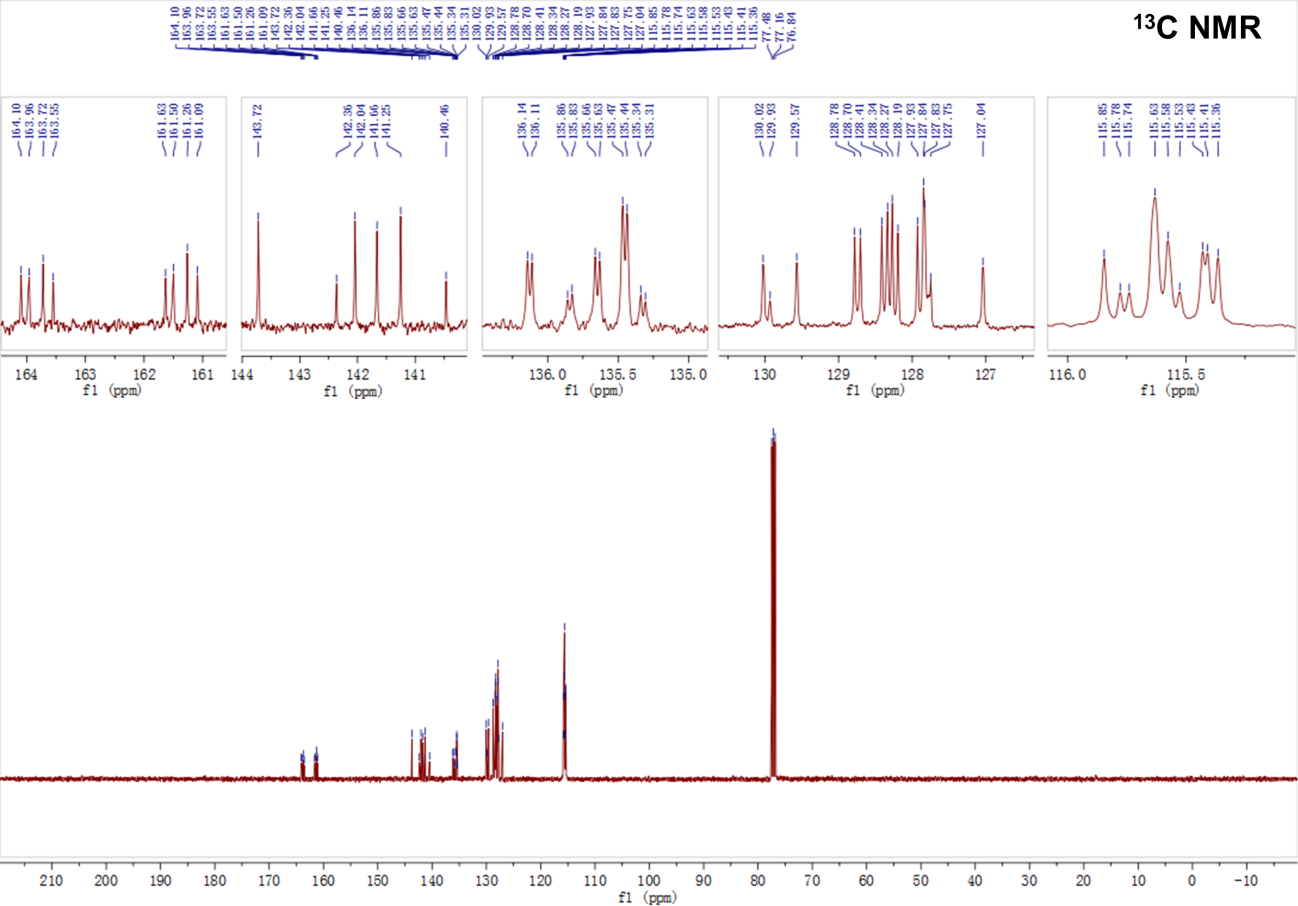
**

**Fig. S21.**  ^1^H NMR and ^13^C NMR spectrum of cyclooctatetraene compounds synthesized by 4-fluorophenylacetylene.


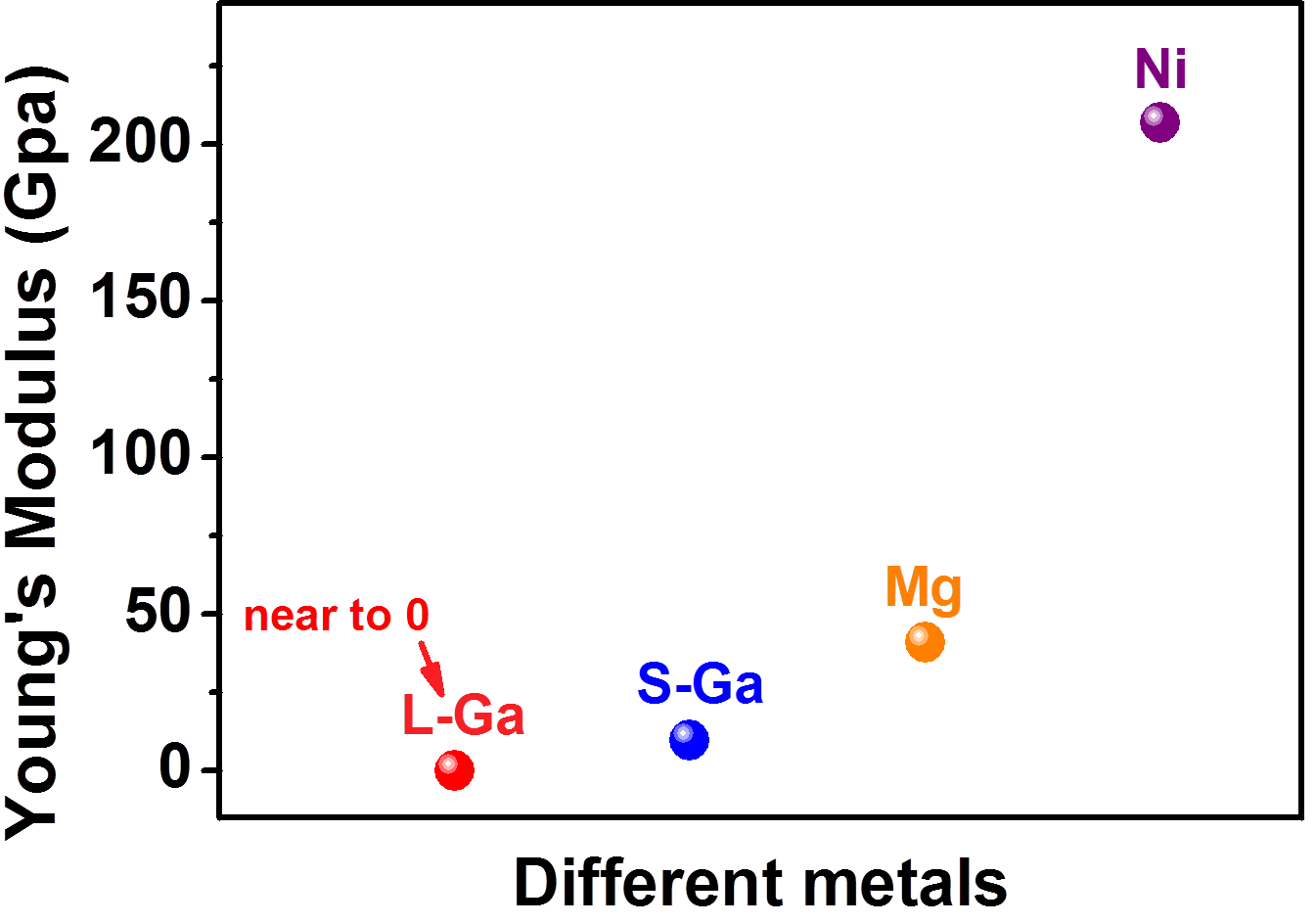


**Fig. S22.** Young’s modulus diagram of L-Ga [[4](#_ENREF_4)], S-Ga [[5](#_ENREF_5)], solid Mg [[6](#_ENREF_6)] and solid Ni [[7](#_ENREF_7)]. Through the analysis in the previous text (Analysis of the distribution of the loss of mechanical energy in a reaction is loaded on the page 5 of Supporting information), the level of energy consumption can be intuitively seen.


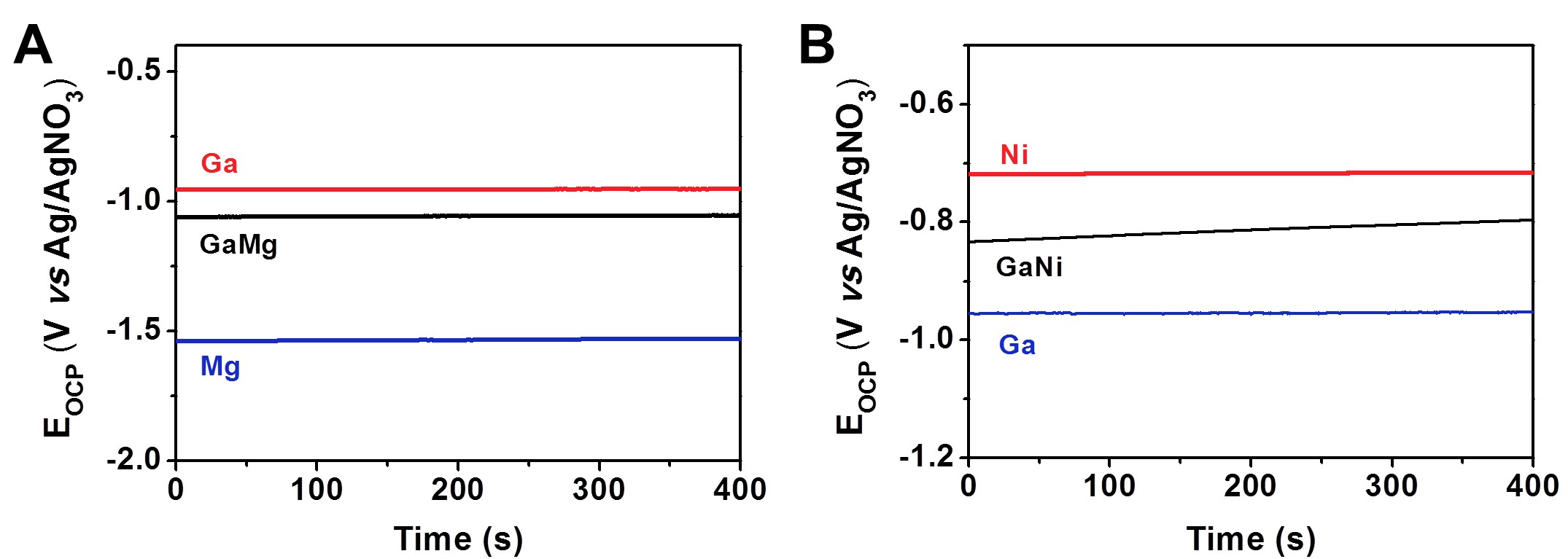


**Fig. S23.** Open-circuit potential tests for Mg, Ni, L-Ga, L-GaMg and L-GaNi. (A) The OCP *vs* time curve with L-Ga, L-GaMg and Mg. (B) The OCP vs time curve with Ni, L-GaNi and L-Ga.

**
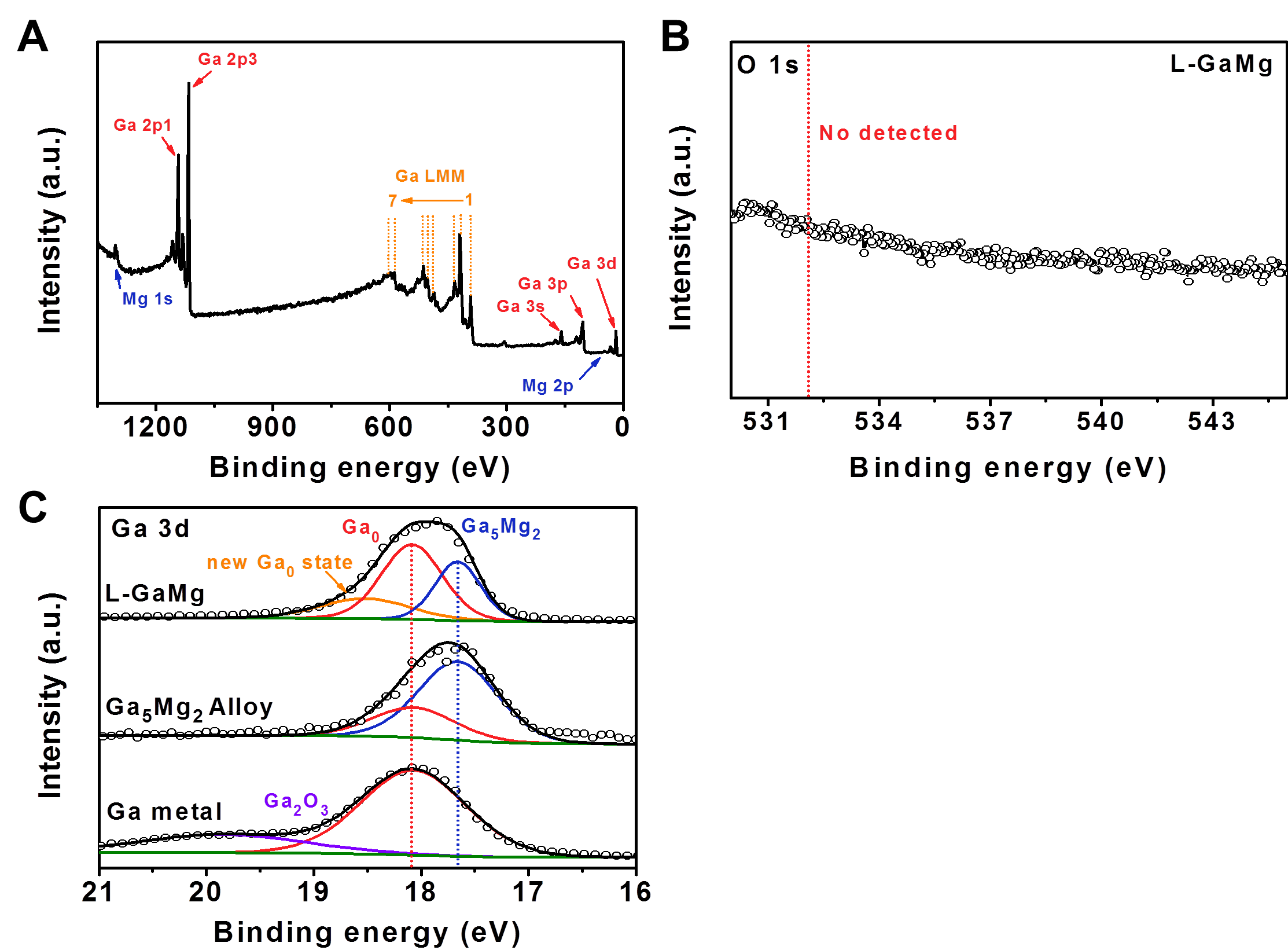
**

**Fig. S24.** XPS characterization of L-GaMg. (A) XPS survey spectra of L-GaMg metal. (B) O1s XPS spectra of L-GaMg metal. (C) Ga3d XPS spectra of L-GaMg metal, Ga_5_Mg_2_ alloy and pure Ga metal.

**
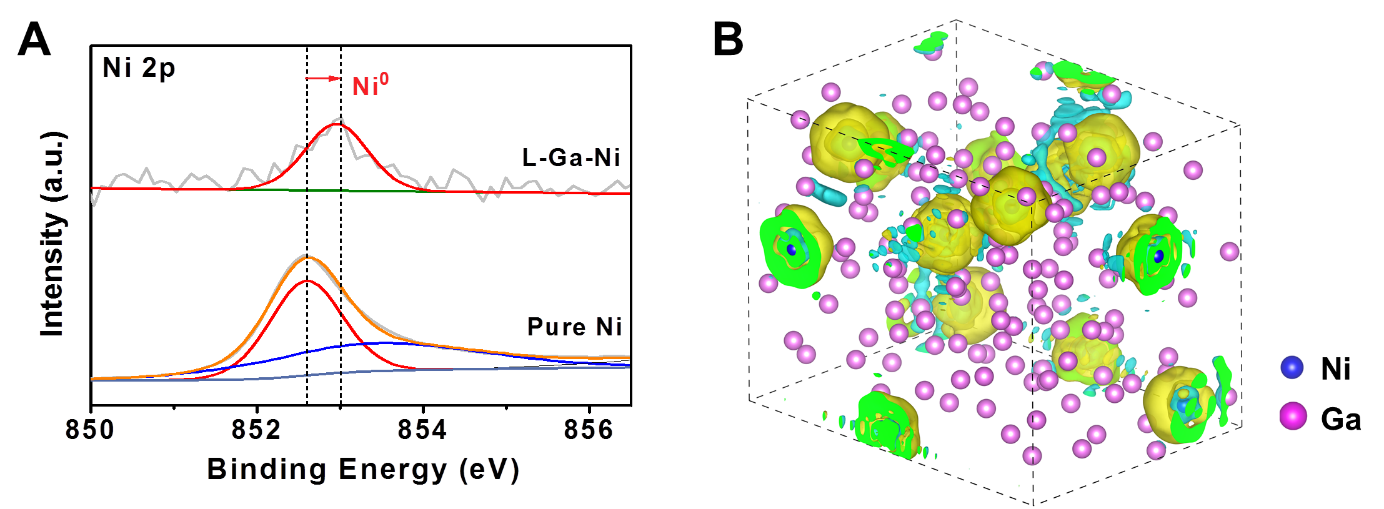
**

**Fig. S25.** (A) Ni 2p XPS spectra of Ni dispersed in liquid gallium. (B) The 3D charge density for liquid Ni-Ga, the gold color represents charge accumulation, while the cyan color represents the charge depletion zone. The isosurface set a uniform value of 0.003 electrons/Å^3^.

**
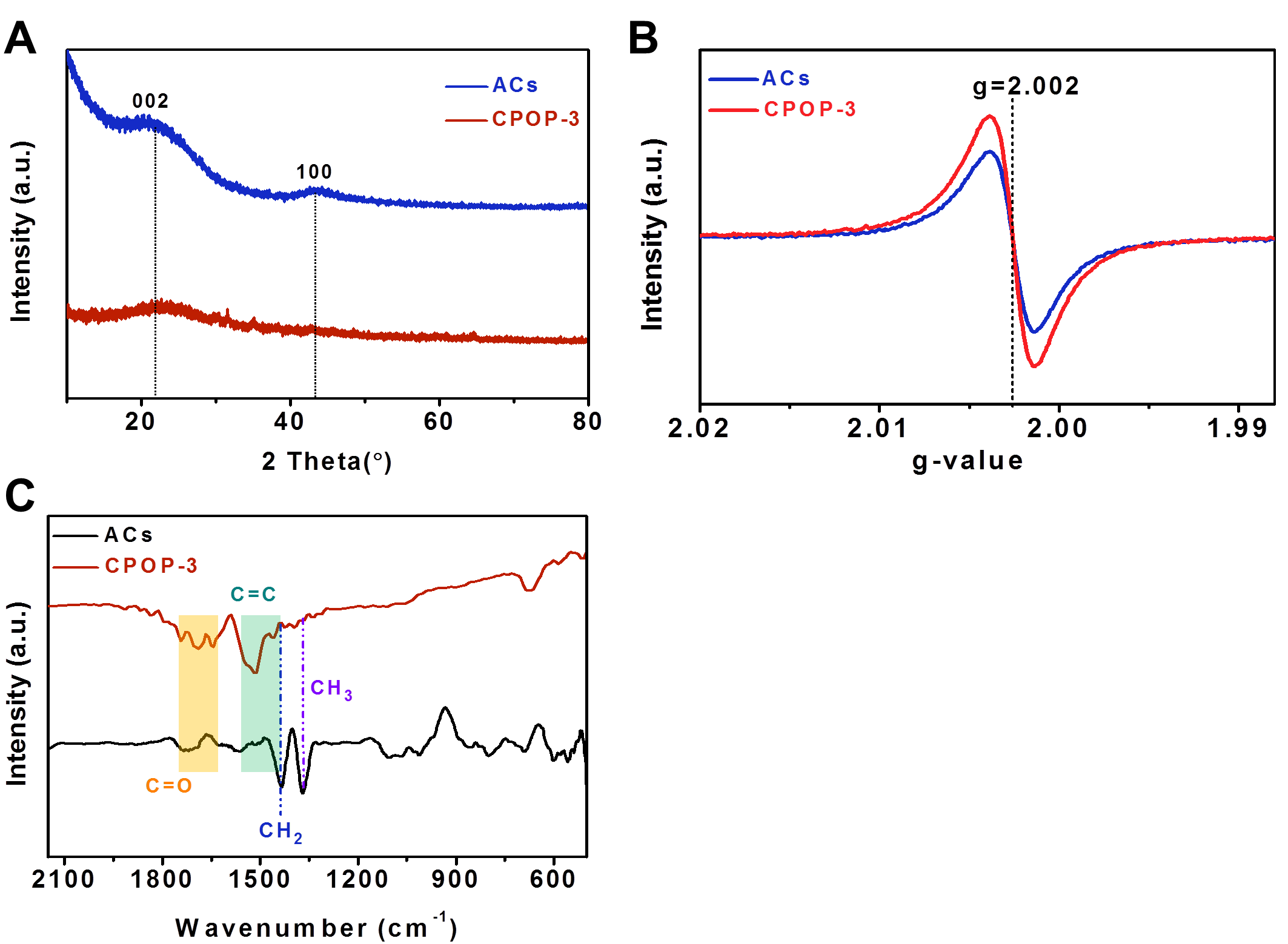
**

**Fig. S26.** Characterization of active carbons (ACs) and CPOP-3. (A) XRD pattern of CPOP-3 and active carbon. (B) EPR spectra of CPOP-3 and active carbon. (C) IR spectrum of CPOP-3 and active carbon.


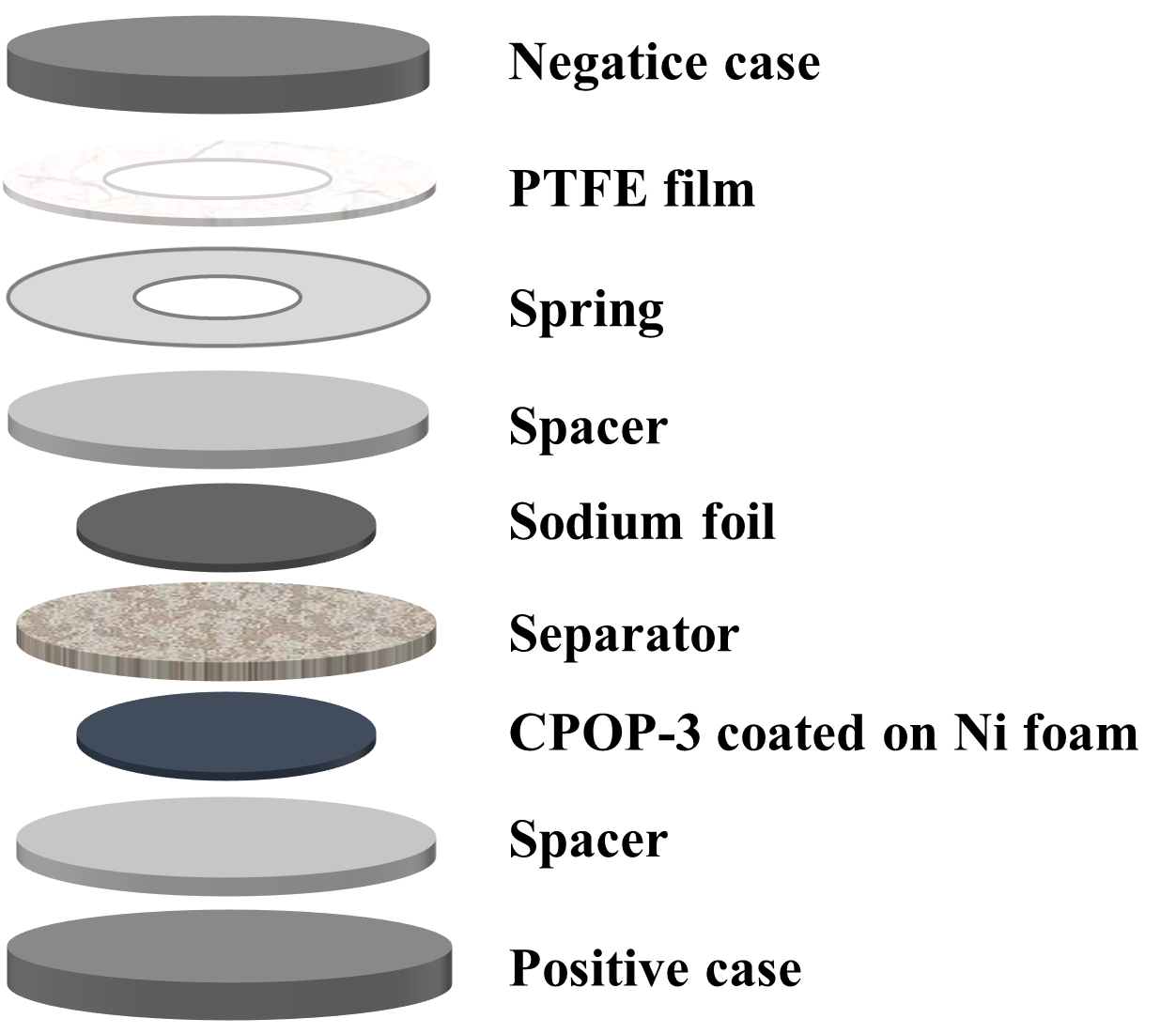


**Fig. S27.** Schematic diagram of Na/Cl_2_ battery assembly structure.

**
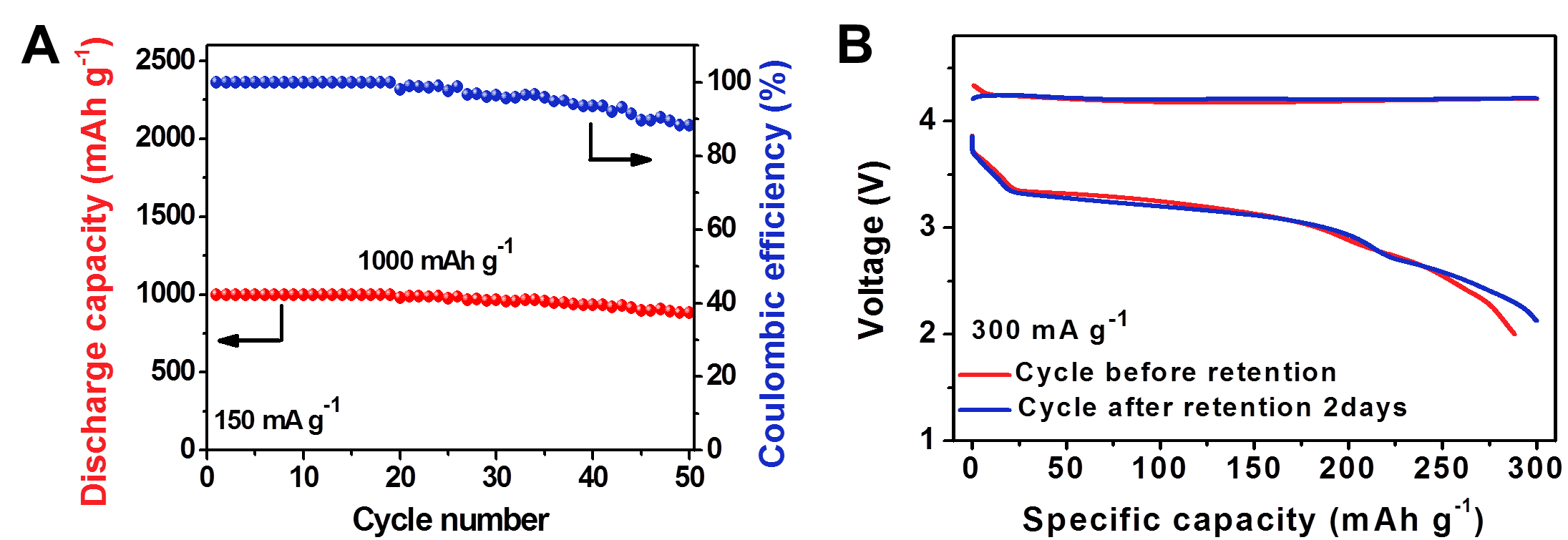
**

**Fig. S28.** Performance of Na/Cl_2_ battery. (A) Cycling performance of Na/Cl_2_ battery when the charging capacity was 1000 mAh g^-1^ at 150 mA g^-1^. (B) Charge–discharge curves of a Na/Cl_2_ battery recorded after discharging the battery post-two-days retention in the charged state.

**Table S1.** Optimization of the conditions for TBPB monomer coupling reaction.

| **Entry** | **Catalyst** | **Atmosphere** | **Time/min** | **Yield (%)^[e]^** |
| --- | --- | --- | --- | --- |
| **1** | Solid Ga | Ar | 20 | No reaction |
| **2** | Liquid Ga | Ar | 20 | 44 |
| **3** | Liquid Ga | Ar | 60 | 58 |
| **4** | Liquid Ga | Ar | 300 | 62 |
| **5** | Liquid Ga | Air | 60 | 56 |
| **6^[a]^** | Liquid Ga | Ar | 60 | No reaction |
| **7^[b]^** | Liquid Ga | Ar | 60 | 41 |
| **8^[c]^** | Liquid Ga | Ar | 60 | 60 |
| **9** | GaInSn | Ar | 60 | 66 |
| **10** | In | Ar | 60 | No reaction |
| **11** | Sn | Ar | 60 | No reaction |
| **12** | Mg | Ar | 60 | No reaction |
| **13** | Zn | Ar | 60 | No reaction |
| **14** | Cu | Ar | 60 | No reaction |
| **15^[d]^** | Liquid Ga | Ar | 600 | No reaction |
| **16** | no | Ar | 60 | No reaction |
| **17^[f]^** | Liquid Ga | Ar | 60 | 65 |
| **18** | Ga_2_O_3_ | Ar | 60 | No reaction |

Reaction condition: A 25 ml ZrO_2_ milling jar with gas outlet/inlet valves and ZrO_2_ balls (4 balls (Ø 10 mm); 14 balls (Ø 5 mm); 14 balls (Ø 2 mm)) were used. Tetrabromo-1,4-benzoquinone (1 mmol) and metal catalyst (4 equiv.) were reacted at speed of 270 rad/min. [a] Metal catalyst (1 equiv. ); [b] Metal catalyst (2 equiv.); [c] Metal catalyst (6 equiv. ); [d] Ultrasonic treatment of 4 equiv. liquid gallium in DMSO solvent. [e] The yield is calculated according to the actual product. [f] at a speed of 360 rad/min.

**Table S2.** The carbon-halogen bond dissociation energy of different halide monomers.

| **Monomer** | **Structure** | **C-X BDE (kJ mol^-1^)** |
| --- | --- | --- |
| 1, 3, 5-tris(bromomethyl)benzene  (**TBB**) | 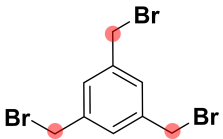 | 211.3[[8](#_ENREF_8)] |
| 2,3,5,6-tetrabromo-1,4-benzoquinone (**TBPB**) | 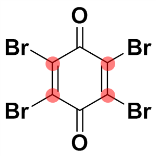 | 276[[9](#_ENREF_9)] |
| 2, 3, 5, 6-tetrachloro-1, 4-benzoquinone  (**TCPBQ**) | 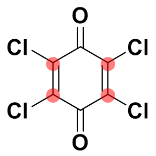 | 338[[9](#_ENREF_9)] |
| Phosphorus oxychloride  (**POC**) | 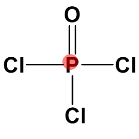 | 345.2[[10](#_ENREF_10)] |
| 2, 4, 6-trichloro-1, 3, 5-triazine  (**TCT**) | 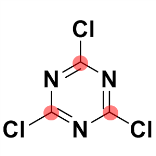 | 363[[11](#_ENREF_11)] |
| Tetrachloroterephthalonitrile  (**TCTPAN**) | 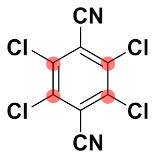 | 365[[12](#_ENREF_12)] |

**Table S3.** Optimization of the conditions for TCTPAN monomer coupling reaction.

| **Entry** | **L-Ga (mmol)** | **Mg powder (mmol)** | **Time/h** | **Yield (%)** |
| --- | --- | --- | --- | --- |
| **1** | 5 | 0 | 3 | No reaction |
| **2** | 0 | 2.5 | 3 | No reaction |
| **3** | 5 | 2.5 | 3 | 72.7 |
| **4** | 5 | 2.5 | 1 | No reaction |
| **5** | 5 | 2.5 | 5 | 84.6 |
| **6** | 5 | 2.5 | 9 | 87.4 |
| **7** | 3 | 2.5 | 5 | 79.0 |
| **8** | 7 | 2.5 | 5 | 92.7 |
| **9** | 0 | 2.5 | 5 | No reaction |
| **10** | 0 | 2.5 | 9 | 48.2 |
| **11** | 5 | 0 | 9 | No reaction |

Reaction condition: A 25 ml ZrO_2_ milling jar with gas outlet/inlet valves and ZrO_2_ balls (4 balls (Ø 10 mm); 14 balls (Ø 5 mm); 14 balls (Ø 2 mm)) were used. Tetrachloroterephthalonitrile monomer (0.6 mmol), varying molar amounts of magnesium powder and liquid gallium were reacted at speed of 270 rad/min. And the reaction atmosphere must be replaced with argon at least three times.

**Table S4.** Optimization of the conditions for [2+2+2+2] cycloaddition of Ethyl propiolate.

| **Entry** | **Ni powder（mol）** | **Liquid Ga（mol）** | **Time (h)** | **Yield (%)** |
| --- | --- | --- | --- | --- |
| **1** | 7.2 | 0 | 3 | 13.6 |
| **2** | 7.2 | 5 | 3 | 37 |
| **3** | 7.2 | 0 | 5 | 23.8 |
| **4** | 7.2 | 5 | 5 | 69.8 |
| **5** | 7.2 | 0 | 9 | 40.1 |
| **6** | 7.2 | 5 | 9 | 74.2 |
| **7** | 7.2 | 1.7 | 5 | 64.8 |
| **8** | 7.2 | 14 | 5 | 74.3 |
| **9** | 3 | 5 | 5 | 15.4 |
| **10** | 0 | 5 | 3 | No reaction |
| **11** | 0 | 5 | 5 | No reaction |
| **12** | 0 | 5 | 9 | No reaction |

Reaction condition: A 25 ml ZrO_2_ milling jar with gas outlet/inlet valves and ZrO_2_ balls (4 balls (Ø 10 mm); 20 balls (Ø 5 mm); 20 balls (Ø 2 mm)) were used. 6 mmol ethyl propiolate, varying molar amounts of Ni powder and liquid gallium were reacted at speed of 270 rad/min. And the reaction atmosphere must be replaced with argon at least three times.

**Movie S1** (separate file). Comparison of metal reactivity between liquid Ga-Mg composite catalyst and pure magnesium particles in warm water (40℃).

**Movie S2** (separate file). Simulation of product concentration changes catalyzed by solid and liquid metal catalyst.

**Movie S3** (separate file). Dynamic motion of magnesium metal dispersed in liquid gallium.

**Movie S4** (separate file). Simulation of dynamic motion of active magnesium and nickel dispersed in liquid gallium.

# References

1. Haley RA, Zellner AR, Krause JA, Guan H, Mack J. Nickel catalysis in a high speed ball mill: a recyclable mechanochemical method for producing substituted cyclooctatetraene compounds. *ACS Sustainable Chem Eng*. 2016;4(5):2464-2469.

2. Chen Z, Lu S, Mao Q, Buekens A, Wang Y, Yan J. Energy transfer and kinetics in mechanochemistry. *Environ Sci Pollut Res*. 2017;24(31):24562-24571.

3. Lu H, Huang X, Li D. Understanding the bond-energy, hardness, and adhesive force from the phase diagram via the electron work function. *J Appl Phys*. 2014;116(17):173506.

4. Yu J, Xia J, Guan X, Xiong G, Zhou H, Yin S, Chen L, Yang Y, Zhang S, Xing Y, et al. Self-healing liquid metal confined in carbon nanofibers/carbon nanotubes paper as a free-standing anode for flexible lithium-ion batteries. *Electrochim Acta*. 2022;425:140721.

5. Yuan B, Zhao C, Sun X, Liu J. Liquid-metal-enhanced wire mesh as a stiffness variable material for making soft robotics. *Adv Eng Mater*. 2019;21(10):1900530.

6. Zhao D, Witte F, Lu F, Wang J, Li J, Qin L. Current status on clinical applications of magnesium-based orthopaedic implants: A review from clinical translational perspective. *Biomaterials*. 2017;112:287-302.

7. Giallonardo JD, Erb U, Aust KT, Palumbo G. The influence of grain size and texture on the Young's modulus of nanocrystalline nickel and nickel-iron alloys. *Philos Mag*. 2011;91(36):4594-4605.

8. Szwarc M, Ghosh BN, Sehon AH. The C-Br bond dissociation energy in benzyl bromide and allyl bromide. *J Chem Phys*. 1950;18(9):1142-1149.

9. Pla FP, Hall CD, Valero R, Pons M. Kinetics and mechanism of the addition of triphenylphosphoniocyclopentadienide to tetrahalo-p-benzoquinones. Part III. The disubstitution of chloranil and bromanil. *J Chem Soc, Perkin Trans. 2*. 1994:2217-2227.

10. Hartley SB, Holmes WS, Jacques JK, Mole MF, McCoubrey JC. Thermochemical properties of phosphorus compounds. *Q Rev Chem Soc*. 1963;17(2):204-223.

11. Nguyen MM, Al-Abdul-Wahid MS, Fontenot KR, Graves EE, Chang S, Condon BD, Grimm CC, Lorigan GA. Understanding the mechanism of action of triazine-phosphonate derivatives as flame retardants for cotton fabric. *Molecules*. 2015;20(6):11236-11256.

12. Chen H, Fan J, Fu Y, DoThanh C, Suo X, Wang T, Popovs I, Jiang D, Yuan Y, Yang Z, et al*.* Benzene ring knitting achieved byambient-temperature dehalogenation via mechanochemical ullmann-type reductive coupling. *Adv Mater*. 2021;33(21):2008685.
